# Early and comprehensive care bundle in the elderly for acute heart failure in the emergency department: study protocol of the ELISABETH stepped-wedge cluster randomized trial

**DOI:** 10.1186/s13063-019-3188-8

**Published:** 2019-01-31

**Authors:** Yonathan Freund, Judith Gorlicki, Marine Cachanado, Sarah Salhi, Vanessa Lemaître, Tabassome Simon, Alexandre Mebazaa

**Affiliations:** 1 Sorbonne Université, Paris, France; 2 Emergency Department, Hôpital Pitié-Salpêtrière, Paris, France; 3 Emergency Department, Hôpital Avicenne, Université Paris 13, Sorbonne Paris Cité, Bobigny, France; 4 Clinical Research Platform, Hôpital Saint-Antoine, Paris, France; 5 Department of Anaesthesiology and Critical Care Medicine, Saint Louis and Lariboisière University Hospitals and INSERM UMR-S 942, Paris, France; 6 Service d’accueil des urgences, Hôpital Pitié-Salpêtrière, 83 boulevard de l’hôpital, 75013 Paris, France

**Keywords:** Elderly, Acute heart failure, Emergency department

## Abstract

**Background:**

Acute heart failure (AHF) is one of the most common diagnoses for elderly patients in the emergency department (ED), with an admission rate above 80% and 1-month mortality around 10%. The European guidelines for the management of AHF are based on moderate levels of evidence, due to the lack of randomized controlled trials and the scarce evidence of any clinical added value of a specific treatment to improve outcomes. Recent reports suggest that the very early administration of full recommended therapy may decrease mortality. However, several studies have highlighted that elderly patients often received suboptimal treatment. Our hypothesis is that an early care bundle that comprises early and comprehensive management of symptoms, along with prompt detection and treatment of precipitating factors should improve AHF outcome in elderly patients.

**Methods/design:**

ELISABETH is a stepped-wedge, cluster randomized controlled, clinical trial in 15 emergency departments in France recruiting all patients aged 75 years and older with a diagnosis of AHF. The tested intervention is a care bundle with a checklist that mandates detection and early treatment of AHF precipitating factors, early and intensive treatment of congestion with intravenously administered nitrate boluses, and application of other recommended treatment (low-dose diuretics, non-invasive ventilation when indicated, and preventive low-molecular-weight heparin). Each center is randomized to the order in which they will switch from a “control period” to an “intervention period.” All centers begin the trials with the control period for 2 weeks, then after each 2-week step a new center will enter the intervention period. At the end of the trial, all clusters will receive the intervention regimen. The primary outcome is the number of days alive and out of the hospital at 30 days.

**Discussion:**

If our hypothesis is confirmed, this trial will strengthen the level of evidence of AHF guidelines and stress the importance of the associated early and comprehensive treatment of precipitating factors. This trial could be the first to report a reduction in short-term morbidity and mortality in elderly AHF patients.

**Trial registration:**

ClinicalTrials.gov, ID: NCT03683212. Prospectively registered on 25 September 2018.

**Electronic supplementary material:**

The online version of this article (10.1186/s13063-019-3188-8) contains supplementary material, which is available to authorized users.

## Background

Acute heart failure (AHF) is a syndrome defined as new-onset or worsening of symptoms and signs of HF, often requiring rapid escalation of therapy and hospital admission. The clinical presentation of AHF typically includes symptoms or signs related to congestion and volume overload rather than to hypoperfusion [1]. Acute heart failure represents 5% of all emergency hospitalizations, and is the most common primary diagnos in patients aged ≥75 years visiting the emergency department (ED) [2, 3]. The EDs are the main entry to the hospital for AHF, with 64% of these admissions being subsequent to an ED visit [4]. This syndrome is reportedly associated with poor outcomes, with a 80% rate of hospital admission, a median length of hospital stay of 10 days and a mortality around 10% at 30 days, and a readmission rate of 25–30% at 30 days [5–7]. Despite a high rate of morbidity and mortality, the management of AHF has not changed for several decades and most clinical studies failed to demonstrate a positive impact of new drugs on patients’ prognosis [8, 9]. European guidelines include the use of diuretics, nitrates, oxygen and non-invasive ventilation (NIV) when indicated along with the treatment of any potential AHF triggers (precipitating factors). However, these guidelines are based on moderate levels of evidence (IB and IIaB), and high-quality randomized controlled trial (RCT) data are lacking [10–12].

In 1998 and 2000, the two cornerstone trials of Cotter et al. provided evidence of the benefits associated with early vasodilator therapy with nitrates, although on a very small sample of patients (less than 200 in total) [13, 14]. Since then, every prospective trial on AHF management failed to report a clinically significant improvement of outcomes. Equipoise remains on many questions regarding the recommended therapeutics: the optimum dose and route of administration of diuretics are not clear, the use of nitrates is also debated, and the benefit of NIV is unclear [15–17]. Despite these controversies, recommendations and guidelines are published by international societies (European Society of Cardiology (ESC), American Heart Association (AHA), etc.) and constitute the basis of our understanding and standard of care [10, 11]. However, a large proportion of elderly patients in AHF do not receive adequate care, including low rates (30–50%) of nitrate therapy [7, 18]. We recently conducted a preliminary analysis in eight French EDs participating in the present ELISABETH trial. For a 7-day period, we evaluated all consecutive patients aged 75 years and older with a diagnosis of AHF in the ED. Among the 73 consecutive AHF patients, 23 patients (32%) had not been investigated for the findings of precipitating factors of AHF (namely infection, acute coronary syndrome (ACS) or atrial fibrillation). In total, only 18 elderly ED patients (23%) were managed according to the existing guidelines [19].

The lack of solid evidence regarding the efficacy of full recommended therapeutic management of AHFS on outcomes may have been caused by several shortcomings that we will address in the present ELISABETH trial:

1. The previous RCTs did not include in their protocol of care the systematic early assessment for precipitating factors, and their subsequent treatment. The main reported triggers are ACS, infection and atrial fibrillation [20, 21]. As the outcomes of AHF patients has been linked with the triggering factors, we make the hypothesis that early (i.e., in the ED) and comprehensive discovery and treatment of these precipitating factors may improve the prognosis [22]
2. The majority of previous RCTs only assessed the impact of single drugs, and not of a comprehensive care bundle. Due to polyfactorial causes of poor outcomes in elderly patients with AHF, we believe that an intervention that focuses only on the administration of a single drug may have a lesser effect than a care bundle
3. The delay between ED arrival and randomization may have been too long: in recent large RCTs, this timeframe varied from 6 to > 24 h [8, 23]. It has, however, been suggested that the introduction of decongestion treatment within hours in the ED is associated with better outcomes [24–26]. In the present study, nitrates and loop diuretics will be given within 1 h of first medical contact in the ED
4. Although the elderly are described as suffering most from AHF, with worse outcomes, specific RCTs in this frail population are lacking (e.g., the recent True-AHF RCT, which evaluated the effect of ularitide infusion, excluded elderly patients) [8]. Thus our trial, focused on older AHF patients, will be, to the best of our knowledge, the first to evaluate the impact of an early intensive approach in this target population

The hypothesis of our trial is that an early and comprehensive care bundle, that associates prompt treatment of congestion with intravenously administered (IV) nitrate boluses and early recognition and treatment of potential precipitating factors, will improve short-term morbidity and mortality.

## Methods/design

The ELISABETH trial (NCT03683212) is a stepped-wedge clinical trial in France [27]. The primary objective of this study is to assess the change in the early (1-month) morbidity and mortality of AHF in elderly patients with the implementation of an early and comprehensive care bundle in the ED.

### Experimental plan of the stepped-wedge design

In this stepped-wedge clinical trial, patients will be recruited in 15 EDs in France, academic and non-academic, rural and urban (Table 1). All clusters will begin the trial with the “control period” where included AHF patients will be routinely managed by the emergency physicians. After a first step of 2 weeks, every 2 weeks, one center will randomly be assigned to switch to the “intervention period” where the ELISABETH care bundle will be implemented. After 32 weeks, all centers will be in the “intervention period” for the four remaining weeks of the trial (Table 2).

**Table 1:** List of investigators and recruiting centers

Name	First name	City	Country	Hospital	Expected recruitment
Freund	Yonathan	Paris	France	Pitié-Salpêtrière	34
Adnet	Frederic	Paris	France	Avicenne	34
Yordanov	Youri	Paris	France	Saint-Antoine	34
Feral	Anne-Laure	Paris	France	HEGP	34
Laribi	Said	Tours	France	CHU Tours	34
Claret	Pierre-Geraud	Nîmes	France	CHU Nîmes	34
Chouihed	Tahar	Nancy	France	CHU Nancy	34
Charpentier	Sandrine	Toulouse	France	CHU Rangueuil	34
Truchot	Jennifer	Paris	France	Lariboisiere	34
Dumas	Florence	Paris	France	Cochin	34
Occelli	Celine	Nice	France	CHU Nice	34
Khellaf	Mehdi	Creteil	France	CHU H Mondor	34
Beaune	Sebastien	Boulogne	France	CHU A Paré	34
Ganansia	Olivier	Paris	France	CH St Joseph	34
Desmettre	Thibaut	Besancon	France	CHU Besancon	34

**Table 2:** Experimental design of the stepped-wedge methodology

	Step 1	Step 2	Step 3	Step 4	Step 5	Step 6	Step 7	Step 8	Step 9	Step 10	Step 11	Step 12	Step 13	Step 14	Step 15	Step 16
Center 1	C	I	I	I	I	I	I	I	I	I	I	I	I	I	I	I
Center 2	C	C	I	I	I	I	I	I	I	I	I	I	I	I	I	I
Center 3	C	C	C	I	I	I	I	I	I	I	I	I	I	I	I	I
Center 4	C	C	C	C	I	I	I	I	I	I	I	I	I	I	I	I
Center 5	C	C	C	C	C	I	I	I	I	I	I	I	I	I	I	I
Center 6	C	C	C	C	C	C	I	I	I	I	I	I	I	I	I	I
Center 7	C	C	C	C	C	C	C	I	I	I	I	I	I	I	I	I
Center 8	C	C	C	C	C	C	C	C	I	I	I	I	I	I	I	I
Center 9	C	C	C	C	C	C	C	C	C	I	I	I	I	I	I	I
Center 10	C	C	C	C	C	C	C	C	C	C	I	I	I	I	I	I
Center 11	C	C	C	C	C	C	C	C	C	C	C	I	I	I	I	I
Center 12	C	C	C	C	C	C	C	C	C	C	C	C	I	I	I	I
Center 13	C	C	C	C	C	C	C	C	C	C	C	C	C	I	I	I
Center 14	C	C	C	C	C	C	C	C	C	C	C	C	C	C	I	I
Center 15	C	C	C	C	C	C	C	C	C	C	C	C	C	C	C	I

Each step lasts for 2 weeks, except for step 1 and step 16 which last for 4 weeks. *I* Intervention, *C* Control

We decided to choose this design for the following reasons:

- As we implement a new protocol, there is a risk of contamination. An emergency physician, who would have already treated patients via the care bundle protocol, would be subsequently influenced by this trial, and could have difficulty in providing the former “standard of care.” Therefore, a randomization at the patient level or a cross-over design would induce bias through contamination
- The present ELISABETH trial focuses on a severe condition, in EDs that are often busy places; therefore, the need for randomization at the patient level could be an impediment to inclusion, and, therefore, limit our ability to recruit consecutive patients
- A cluster, stepped-wedge design prevents contamination that could arise from a cluster cross-over design, as centers will first be allocated to standard care before implementing the intervention. Furthermore, a stepped-wedge design would also prevent a potential “period effect” that could have resulted from a simple before/after design

### Selection of participants

The ELISABETH trial focuses on elderly patients with AHF in the ED. Therefore, all patients aged 75 years and over, affiliated to French social security with a diagnosis of AHF in the ED, defined by the association of:

- At least one of the following symptoms: acute or worsening dyspnea, orthopnea
- *and* at least one of the following:
  - ○ Bilateral pulmonary râles or peripheral edema
  - ○ Signs of pulmonary congestion on chest radiography or cardiac echography
  - ○ Increased natriuretic peptides (brain natriuretic peptide (BNP) or NT-proBNP)

A written informed consent signed by the patient will be necessary prior to inclusion. If the patient is unable to consent, then the physician will seek consent from a trustworthy person, family member or close relative. If none are available, the physician will be able to proceed to an emergency inclusion and then the written informed consent will be signed by the patient (if need be by a trustworthy person, family member or close relative) as soon as possible (article L1122-1-2 of the French Public Health Code).

Patients are excluded if they have any of the followings:

- Other obvious cause of acute illness (severe sepsis, ST-elevation myocardial infarction)
- Systolic blood pressure less than 100 mmHg
- Any contra-indication to nitrates (severe mitral or aortic stenosis, or severe aortic regurgitation)
- Known chronic kidney injury on dialysis
- Time from ED entrance to inclusion > 6 h
- Patient under legal protection measure (tutorship or curatorship) and patient deprived of freedom

### Trial objectives and outcomes

The main objective of the present trial is to compare the efficacy of an early and comprehensive management strategy of AHF in elderly patients to the usual care on morbi-mortality at 30 days. Our primary endpoint is the number of days alive and out of hospital at 30 days after the index ED visit. This endpoint is considered as relevant by the group of experts of the ESC [28]. In their consensus paper, the experts stated that although mortality should be captured, repeated hospitalizations should also be recorded. Especially in elderly patients, where the rate of readmission to the ED and rehospitalization is elevated: up to 40% of heart failure admissions to the hospital are actually repeated admission for recurrence of symptoms within 30 days of a previous AHF event [3, 6]. We chose the timeframe of 30 days because a shorter timeframe would not catch recurrence and morbidity, and a longer timeframe would catch events that are more likely linked to chronic morbidity of the patients than to the AHF syndrome [22, 28, 29].

The primary endpoint (days alive and out of hospital at day 30) will be measured at the end of the 30-day follow-up period, either by hospital visit or phone interview, and medical chart review. Vital status, date of death and date of discharge will be collected.

A death during the follow-up period will correspond to 0. An ED visit will correspond to “1 day” at the hospital. For example, a patient not admitted (at day 0), with no return visit to the hospital, and alive at day 30 will have 30 days alive and out of hospital. A patient who is admitted (at day 0) and stays 8 days in the hospital before being discharged and has no readmission and no return visit to the ED would have “22 days alive and out of hospital at 30 days.” A patient who is admitted and dies at 13 days, either at home or in hospital will score 0. A patient who is admitted for 10 days, discharged home for 5 days then admitted at day 16 for 15 days will have 5 days alive and out of hospital (namely days 11, 12, 13, 14 and 15).

The secondary endpoints include:

- 30-day all-cause mortality
- 30-day cardiovascular mortality
- Hospital readmission at 30 days
- Length of in hospital stay truncated at 30 days
- Changes of more than twofold in creatinine level from inclusion to day 30 or to discharge, whichever comes first

Creatinine will be measured at day 0 in the ED, and at discharge day or day 30, whichever comes first.

### Description of the intervention

This is an intervention study, where the intervention comprises the application of recommendations and guidelines for the management of AHF, ACS and infection.

Control period: Acute heart failure standard therapy:

- Treatments are given at the discretion of the treating emergency physician
  The guidelines and standard of care will be recalled to the emergency physicians at the beginning of the trial in each center when the control period will start.

**Fig. 1:**
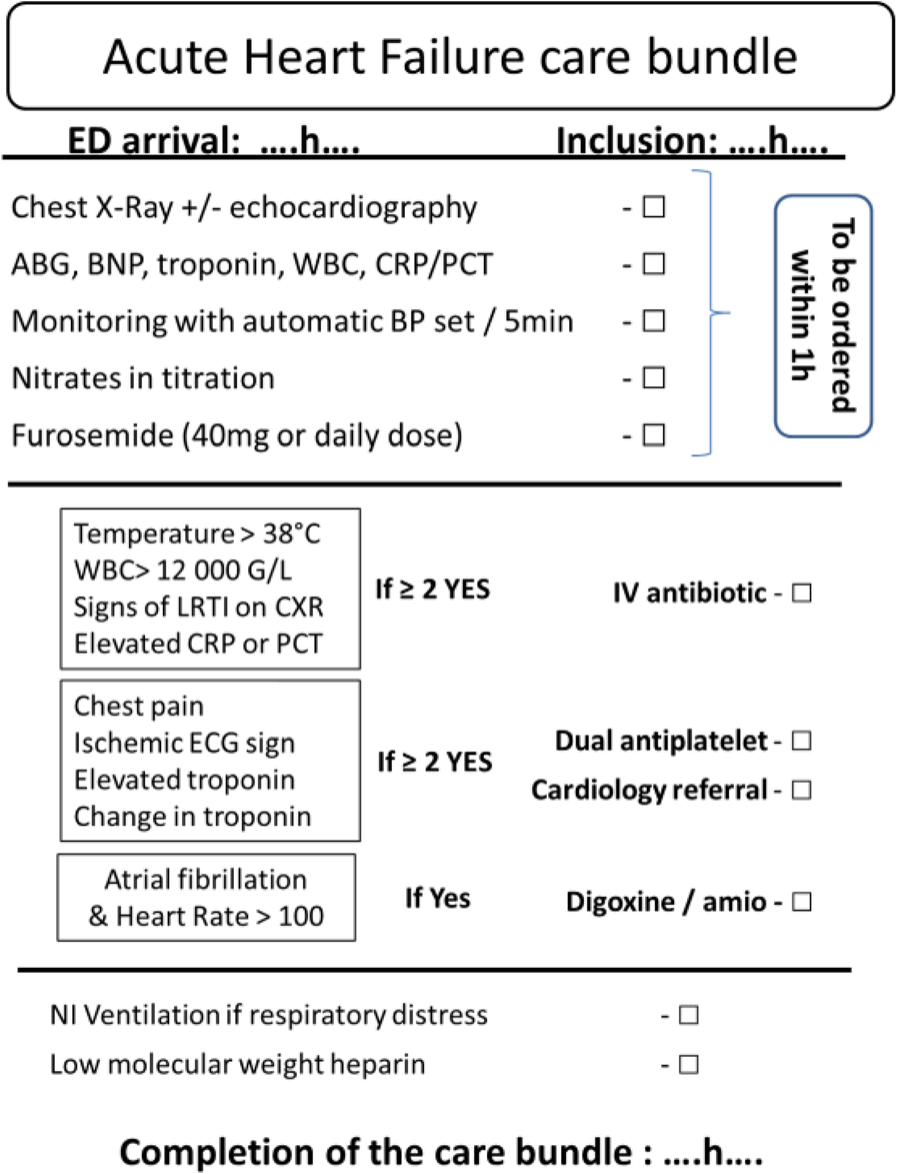
Handover sheet with checklist for acute heart failure (AHF) management

Intervention period: Early intensive care bundle:

The care bundle comprises a list of items to follow and tick on a handover checklist (Fig. 1) within 4 h of ED management:

- Treatment of the congestion: (*international guidelines and recommendations* [10, 11])
  ○ 40 mg of IV furosemide (or usual daily dose) if not already given pre-hospital
  ○ IV nitrates given in boluses of 3 mg every 5 min. After 1 h of bolus titration, then continuous infusion with an hourly dose of at least half of the total given during the first hour of nitrate administration. Blood pressure (BP) will be monitored every 5 min during the titration (then hourly), and nitrates will be discontinued if BP drops < 100 mmHg
- Treatment of precipitating factors:
  ○ Administration of antibiotic therapy (accordingly to local guidelines amoxicillin and clavulanic acid in most cases) in the presence of at least two of the following: fever > 38 °C, leucocytes > 12,000 G/L, radiological signs suggestive of lower respiratory tract infection or elevated C-reactive protein (CRP) or procalcitonin (PCT)
  ○ Administration of dual antiplatelet therapy and transfer to cardiac intensive care unit in the presence of at least two of the followings: chest pain, ischemic signs on an electrocardiogram (ECG), elevated troponin concentration or change in troponin concentration. These patients will be transferred for coronary angiography if indicated by the cardiologist, as recommended [30]
  ○ In case of atrial fibrillation: administration of heparin, heart rate control strategy (digoxin or amiodarone as indicated) to reduce heart rate under 100 bpm, early admission to a cardiac intensive care unit if elevated troponin is associated
- NIV if respiratory distress with hypercapnia and pH < 7.35 in the absence of any contraindication [11]
- Preventive low-molecular-weight heparin (LMWH) if there is no pre-existing anticoagulation therapy [11]

All treatments will be initiated in the ED, and their continuation or discontinuation will be evaluated by the treating physician during the subsequent hospital stay.

### Statistical analysis

No interim analysis is planned. Analysis will be performed at the end of the study after data review and freezing of the data base. Analyses will be performed using SAS® software (version 9.3 or updated version). Principal analysis will be realized according to the intention-to-treat (ITT) principle.

Baseline patient characteristics will be considered at both with the cluster (center) and patient level. For the center level, characteristics at the beginning of the study will be described (there are no expected changes between the two periods for cluster characteristics). Baseline characteristics of patients will be described globally and according to the period. Continuous variables will be summarized using descriptive statistics, i.e., number of subjects, mean, standard deviation (SD), median, inter quartile range, minimum and maximum. Qualitative variables will be summarized by frequency and percentage.

The number of days alive and out of hospital will be calculated based on date of admission, vital status, date of death and date of discharge will be collected.

This primary endpoint will be analyzed using a linear-regression mixed model with a random effect for each cluster, considered fixed effects will be: strategy and, for the stepped-wedge design, time representing each step. In case of non-normal distribution of the interest variable, a transformation could be realized.

All-cause mortality at 30 days, cardiovascular mortality at 30 days and hospital readmission at 30 days will be compared between groups by using Pearson’s chi-square test or Fisher’s exact test.

If possible, a generalized linear-regression mixed model with Poisson distribution will be performed. If the number of events is sufficient, a generalized linear-regression mixed model using the logit link will be performed. The length of stay in hospital in days will be compared between the two periods by using Student’s *t* test or the Wilcoxon rank-sum test as needed. If possible, a linear-regression mixed model will be performed. A random effect for each cluster will be considered and considered fixed effects will be: strategy and, for the stepped-wedge design, time representing each step. In case of non-normal distribution of the interest variable, a transformation could be realized. The percentage of patients with a change of more than twofold in creatinine between inclusion and 30 days will be compared between groups by using Pearson’s chi-square test or Fisher’s exact test. If possible, a generalized linear-regression mixed model with Poisson distribution will be performed. If the number of events is sufficient, a generalized linear-regression mixed model using the logit link will be performed.

A second analysis will be performed on the per-protocol population.

All tests will be performed with an alpha set at 5%.

### Sample size calculation

From our previous cohort, the mean number of days alive and out of hospital at 30 days was 14 ± 9. To be clinically relevant, we estimate that the new approach should increase this endpoint of 3 days at least (a relative increase of 20%). With a power of 80% and alpha = 5%, 283 patients are needed to be included. Considering the stepped-wedge design and after specification of the following elements: 15 clusters, intra-cluster correlation (ICC) = 0.0001, the design effect is estimated at 1.609, increasing to 454 subjects who need to be included. Taking into account 10% of non-evaluable patients, it is necessary to include 500 patients – two per cluster for each 2-week period (Additional file 1).

## Discussion

Despite a small improvement in the outcomes of elderly patients admitted for AHF within the past decades, its morbidity and mortality remains severe with a 10% rate of 30-day mortality, and 25–30% of early readmission rate [6, 7, 18, 31]. In the majority of cases, treatment can be initiated in the ED. However, many studies have shown that a majority of these patients are still not receiving recommended therapies in the ED – either for AHF per se, or for precipitating factors of AHF, especially ACS or infection [7, 16, 18].

In this context, there is an urgent need for a multidisciplinary management program for patients with AHF in the ED and following ED care to ensure better results and adherence [32]. The great outcome improvements provided by early treatment in the ED have long been established for other pathologies (e.g., sepsis, myocardial infarction). Unfortunately, AHF has not been considered with this regard until recently. Some reports suggest the importance of time to introduce therapy in AHF. Data derived from the ADHERE registry indicate that early treatment (< 6 h) in EDs would create a positive impact by decreasing in-hospital mortality and morbidity rates (unadjusted OR for in-hospital mortality 0.77, adjusted OR 0.87 (95%CI [0.76–0.96]) [24]. Very recently, in their large prospective observational study, Matsue et al. reported a significant decreased mortality in AHF following the initiation of decongestion therapy within 1 h in the ED (OR for in-hospital mortality of 0.39 (95%CI [0.20–0.76]) [26].

As expressed by Januzzi and Felker in a recent editorial, “*the failure of novel therapies for AHF requires us to make better use of what we already have. A systematic approach would allow an optimal management of acute HF, and in turn could finally improve outcomes*” [33]. If our hypothesis is confirmed, our trial of this early intensive care bundle will be the first RCT to show a significant reduction in short-term morbidity and mortality in elderly AHF patients, similar to what was achieved for sepsis (with a 15% absolute reduction of in-hospital mortality) [34, 35].

Lastly, it can be stressed that the observed high rate of deviation from the guidelines may be caused in part by their low level of evidence. A positive outcome of the introduction of a care bundle based on these recommendations would increase physician adherence and patient outcomes.

## Trial status

This is adapted from version v4–0.2018_11_28 of the ELISABETH protocol (Additional file 2).

Inclusion will start on 10 December 2018 and end on 19 August 2019.

## Additional files

Additional file 1: Standard Protocol Items: Recommendations for Interventional Trials (SPIRIT) 2013 Checklist: recommended items to address in a clinical trial protocol and related documents. (DOC 121 kb)

Additional file 2: This is the full ELISABETH protocol in its 4.0 version of 28 November 2018. (DOCX 356 kb)

## Abbreviations

AHF: Acute heart failure
BNP: Brain natriuretic peptide
BP: Blood pressure
ED: Emergency department
ICC: Intra-cluster correlation
ITT: Intention-to-treat
LMWH: Low-molecular-weight heparin
NIV: Non-invasive ventilation
RCT: Randomized controlled trial
