# Supplementary material for: Early and comprehensive care bundle in the elderly for acute heart failure in the emergency department: study protocol of the ELISABETH stepped-wedge cluster randomized trial
Source: Trials. 2019 Jan 31;20:95. doi: 10.1186/s13063-019-3188-8 (PMC6357377; doi:10.1186/s13063-019-3188-8)
Supplement: Supplementary file 2 — This is the full ELISABETH protocol in its 4.0 version of 28 November 2018. (DOCX 356 kb) [file 13063_2019_3188_MOESM2_ESM.docx]

| Early and comprehensive care bundle in elderly for acute heart failure:  a stepped wedge cluster randomized trial  **ELISABETH** |
| --- |

MINIMAL RISKS AND CONSTRAINTS HUMAN RESEARCH STUDY

Version N°4-0 of 28/11/2018

Sponsor code: K170918J /IDRCB: 2018-A01139-46

Coordinating Investigator: Yonathan FREUND

Service d’accueil des urgences

Hôpital Pitié-Salpêtrière, APHP

06.63.54.90.17 – yonatman@gmail.com

Scientific Director: Alexandre MEBAZAA

Département d’anesthésie-réanimation

Hôpital Lariboisière, APHP

01.49.95.80.71 – alexandre.mebazaa@aphp.fr

Sponsor: AP-HP and by delegation: Clinical Research and Innovation Delegation(DRCI)

Hôpital Saint-Louis

1, avenue Claude Vellefaux

DRCI head office project advisor: Anne RADENNE

01.40.27.55.73

anne.radenne@aphp.fr

Entity responsible

Pr T Simon for logisitic coordination, data management and statistical analysis of the trial:

Plateforme de recherché Clinique de l’Est-Parisien (URC-Est-CRC-Est-CRB-UPMC)

Hôpitaux Universitaires Paris Est (AP-HP)

Hôpital Saint-Antoine

Tel. 01 71 97 05 63

Email tabassome.simon@aphp.fr

Clinical Research and Innovation Delegation (DRCI)

Hôpital Saint Louis 75010 PARIS

**PROTOCOL SIGNATURE PAGE**

MINIMAL RISKS AND CONSTRAINTS HUMAN RESEARCH STUDY

Protocol code number: K170918J / IDRCB: 2018-A01139-46

Title: Early and comprehensive care bundle in elderly for acute heart failure:

a stepped wedge cluster randomized trial-ELISABETH

Version N°4-0 of 23/11/2018

The research is carried out in accordance with the current version of the protocol, with GCP and with all statutory and regulatory requirements.

| Coordinating Investigator: |  |
| --- | --- |
| Yonathan FREUND  Service d’accueil des urgences  Hôpital Pitié-Salpêtrière, APHP  Paris | Date: ……………/………/………..  Signature: |
| Sponsor |  |
| Assistance Publique-Hôpitaux de Paris  ClinicalResearch and InnovationDepartment  Hôpital Saint Louis  1 avenue Claude Vellefaux  75010 PARIS | Date: ……………/………/………..  Signature: |
|  |  |

The study was approved by the CPP of “SUD-OUEST & OUTRE-MER II” on 07 September 2018.

**TABLE OF CONTENTS**

[1 SUMMARY 5](#_Toc520797800)

[2 SCIENTIFIC JUSTIFICATION FOR THE RESEARCH 8](#_Toc520797801)

[2.1 CURRENT KNOWLEDGE 8](#_Toc520797802)

[2.2 Hypotheses for the research 10](#_Toc520797803)

[2.3 Description of the population of research participants and justification for the choice of participants 10](#_Toc520797804)

[2.4 Interventions and products which will be performed or used as standard 10](#_Toc520797805)

[2.5 Interventions added for the research 10](#_Toc520797806)

[2.6 Summary of the known and foreseeable benefits and risks for the participants 11](#_Toc520797807)

[3 OBJECTIVES OF THE RESEARCH 11](#_Toc520797808)

[3.1 Main objective of the research 12](#_Toc520797809)

[3.2 Secondary objectives 12](#_Toc520797810)

[4 Description of the research 12](#_Toc520797811)

[4.1 Primary endpoint 12](#_Toc520797812)

[4.2 Secondary endpoints 12](#_Toc520797813)

[5 Research methodology 13](#_Toc520797814)

[5.1 Design of the study 13](#_Toc520797815)

[5.2 Number of participating sites 14](#_Toc520797816)

[5.3 Avoiding and reducing bias 14](#_Toc520797817)

[6 Procedure for the research 14](#_Toc520797818)

[6.1 Schedule for the study 15](#_Toc520797819)

[6.2 Expected length of participation, chronology and duration of the study 16](#_Toc520797820)

[6.3 Table or diagram summarizing the chronology of the research 18](#_Toc520797821)

[6.4 Distinction between standard care and research 18](#_Toc520797822)

[6.5 Biological samples 21](#_Toc520797823)

[7 ELIGIBILITY CRITERIA 21](#_Toc520797824)

[7.1 Inclusion criteria 21](#_Toc520797825)

[7.2 Exclusion criteria 21](#_Toc520797826)

[7.3 Enrolment procedure 22](#_Toc520797827)

[7.4 Inclusion rate 24](#_Toc520797828)

[8 Termination and exit rules 24](#_Toc520797829)

[8.1 Criteria and procedure for premature withdrawals and exits from the study 24](#_Toc520797830)

[9 EFFICACY ASSESSMENt 25](#_Toc520797831)

[9.1 Description of parameters for assessing efficacy endpoints 25](#_Toc520797832)

[9.2 Anticipated methods and timetable for measuring, collecting and analyzing the efficacy data 26](#_Toc520797833)

[10 SAFETY 26](#_Toc520797834)

[11 Specific committee for the study 26](#_Toc520797835)

[11.1 Steering Committee 26](#_Toc520797836)

[12 data management 26](#_Toc520797837)

[12.1 Data collection 27](#_Toc520797838)

[12.2 Identification of data recorded directly in the CRF which will be considered as source data 27](#_Toc520797839)

[12.3 Right to access source data and documents 27](#_Toc520797840)

[12.4 Data processing and storage of research documents and data 28](#_Toc520797841)

[12.5 Ownership of the data 29](#_Toc520797842)

[13 statistical aspects 29](#_Toc520797843)

[13.1 Description of statistical methods to be used including the timetable for the planned interim analyses 29](#_Toc520797844)

[13.2 Principal criterion analysis 29](#_Toc520797845)

[13.3 Secondary evaluation criteria 30](#_Toc520797846)

[13.4 Calculation hypotheses for the number of subjects required and the result 30](#_Toc520797847)

[13.5 Anticipated level of statistical significance 30](#_Toc520797848)

[13.6 Statistical criteria for termination of the study 31](#_Toc520797849)

[13.7 Method for taking into account missing, unused or invalid data 31](#_Toc520797850)

[**13.8** **Management of modifications made to the analysis plan for the initial strategy** 31](#_Toc520797851)

[13.9 Choice of individuals to include in the analyses 31](#_Toc520797852)

[14 QUALITY CONTROL AND ASSURANCE 31](#_Toc520797853)

[14.1 General organization 31](#_Toc520797854)

[14.2 Case report forms 33](#_Toc520797855)

[14.3 Management of non-compliances 33](#_Toc520797856)

[14.4 Audits 33](#_Toc520797857)

[14.5 Principal Investigator's declaration of responsibility 33](#_Toc520797858)

[15 ETHICAL AND LEGAL CONSIDERATIONS 34](#_Toc520797859)

[15.1 Methods for informing and obtaining consent from the research participants 34](#_Toc520797860)

[15.2 Prohibition of concomitant clinical studies participation and exclusion period after the study, if applicable 35](#_Toc520797861)

[15.3 Compensation for participants 35](#_Toc520797862)

[15.4 Registration on a national register of clinical research participants 36](#_Toc520797863)

[15.5 Legal obligations 36](#_Toc520797864)

[15.6 Request for approval from the CPP 36](#_Toc520797865)

[15.7 Informing the ANSM 36](#_Toc520797866)

[15.8 Declaration of compliance with the MR 001 "Reference Method" 36](#_Toc520797867)

[15.9 Modifications to the study 36](#_Toc520797868)

[16 Funding and Insurance 36](#_Toc520797869)

[16.1 Sources of monetary support 37](#_Toc520797870)

[16.2 Insurance 37](#_Toc520797871)

[17 Publication rules 38](#_Toc520797872)

[17.1 Mention of AP-HP affiliation for projects sponsored by AP-HP 38](#_Toc520797873)

[17.2 Mention of the sponsor AP-HP (DRCI) in the acknowledgements of the text 38](#_Toc520797874)

[17.3 Mention of the funder in the acknowledgements of the text 38](#_Toc520797875)

[18 References 38](#_Toc520797876)

[19 List of addenda 42](#_Toc520797877)

# SUMMARY

| Full title | Early and comprehensivecare bundle in elderly for acute heart failure: the ELISABETH stepped wedge cluster randomized trial |
| --- | --- |
| Acronym | Elisabeth |
| Coordinating Investigator | Yonathan FREUND |
| Scientific Director (if applicable) | Alexandre MEBAZAA |
| Sponsor | Assistance Publique-Hôpitaux de Paris |
| Scientific justification | Acute heart failure (AHF) is one of the most common diagnoses for elderly patients in the emergency department (ED), with an admission rate higher than 80% and 1-month mortality around 10%. There is scarce evidence of any clinical added value of a specific treatment to improve outcomes, and European guidelines for the management of AHF are based on moderate levels of evidence, due to the lack of randomized controlled trials.  Recent reports suggest that the very early administration of full recommended therapy may decrease mortality. However, several studies highlighted that elderly patients often received suboptimal treatment: For example, less than a third of them received nitrates therapy while it is recommended. Furthermore, a recent preliminary study reported that only 50% of them are assessed for precipitating factors – although it has been reported that precipitating factors are independently associated with mortality.  Our hypothesis is that an early care bundle that comprises early and comprehensive management of symptoms, along with prompt detection and treatment of precipitating factors should improve AHF outcome in elderly patients. |
| Main objective and primary endpoint | Compare the efficacy of an early and comprehensive strategy in AHF elderly patients to the usual care on morbidity and mortality at 30 days.  Primary endpoint: number of days alive and out of hospital at 30 days after ED visit.  The experts of the European Society of Cardiology has recently considered this endpoint as relevant and adapted (see p. 13). |
| Secondary objectives and endpoints | To evaluate the effect of AHF management on early  morbidity and mortality.  Secondary endpoints:  • 30-day cardiovascular death  • 30-day all causes death  • Hospital readmission at 30 days  • Length of stay in hospital  • Changes of more than 2 fold in creatinine level from inclusion to day 30 or to discharge whichever comes first. |
| Design of the study | Stepped wedge randomised trial (see p. 14)  The 15 participating centers will first be assigned to the “control period” for 4 weeks. Then, every 2 weeks, one center will be randomized to switch to the “intervention period”. After 32 weeks, all centers will then be in the “intervention group” for the last 4-week period of the trial. |
| Population of study participants | Patients aged ≥75 years |
| Inclusion criteria | Patients aged 75 years and older admitted to the emergency department with a diagnosis of AHF determined by the emergency physician, based on the presence of:   - at least one of the following symptoms : acute, or worsening of dyspnea, orthopnea - One or more of the followings: pulmonary rales, peripheral edema, a chest radiograph or transthoracic echocardiography showing pulmonary vascular congestion signs, increased natriuretic peptides (BNP or NT-pro-BNP). - Patients affiliated to French social security (“AME excepted”) - Written informed consent signed by the patient / the trustworthy person / family member / close relative, or inclusion in case of emergency and written informed consent will been signed by the patient (if need be by trustworthy person, family member or close relative) as soon as possible(article L1122-1-2 of the French Public Health Code) |
| Exclusion criteria | Patients are excluded if they have any of the followings:   - other obvious cause of acute illness (severe sepsis, ST elevation Myocardial infarction) - systolic blood pressure less than 100mmHg - severe mitral or aortic stenosis, or severe aortic regurgitation - known chronic kidney injury on dialysis - Time from ED entrance to inclusion > 6h - Patient under legal protection measure (tutorship or curatorship) and patient deprived of freedom |
| Interventions or product under investigation | NA |
| Comparator arm | NA |
| Other interventions added by the study | The intervention will not test any particular drug or medical disposal, but rather the application of the international guidelines (ESC, EUSEM, AHA) through the implementation of a protocol of care. The care bundle of early care with a checklist that includes the following checklist:   - Detection and treatment of AHF precipitating factors (infection, acute coronary syndrome or atrial fibrillation) - Treatment of congestion, including a protocol of nitrates titration, and low dose furosemide, as recommended by international guidelines - Non-invasive ventilation if indicated, as recommended by international guidelines - Preventive low molecular weight heparin, as recommended by international guidelines   During the control period, the standard of care will be treatment as usual, left at the discretion of the treating emergency physician |
| Expected benefits for the participants and for society | We anticipate a reduction in length of hospitalisation and rate of readmission. The intervention is aimed at increasing the number of days alive and at home in the first month after inclusion. This benefit for the patient should be associated with a reduction in the allocation of hospital resource. |
| Minimal risks and constraints added by the study | Minimal risk, as only recommended treatments will be administered  The intervention will not test any particular drug or medical disposal, but rather the application of the international guidelines (ESC, EUSEM, AHA) through the implementation of a protocol of care.  Validated treatment will be randomly attributed to a group of patients (List of interventions listed in the article L1122-1-2 of the French Public Health Code, and corresponding to a Minimal risks and constraints) |
| Scope of the study | Emergency care and acute heart failure |
| Number of participants included | 500patients |
| Number of sites | 15 centers in France  If one center withdraw from the trial before its start, we will invite another ED to participate (2 centers are listed as willing to participate if possible) |
| Schedule for the study | - inclusion period: 36 weeks - participation period (treatment + follow-up): 30 days - total duration: 36 weeks and 30 days |
| Number of enrolments expected per site and per month | 4 |
| Statistical analysis | No interim analysis is planned. Analysis will be performed at the end of the study after data review and freezing of data base according to ITT principle and with regard to cluster level randomisation. |
| Sources of monetary support | French ministry of health |

# SCIENTIFIC JUSTIFICATION FOR THE RESEARCH

## CURRENT KNOWLEDGE

Acute heart failure (AHF) is a syndrome defined as new-onset or worsening of symptoms and signs of HF,often requiring rapid escalation of therapy and hospital admission. The clinical presentation of AHF typically includes symptoms or signs related to congestion and volume overload rather than to hypoperfusion.^1^ Acute heart failure represents 5% of all emergency hospitalizations, and is the most common primary diagnosis in patients ≥75 years visiting the ED.^2,3^ The ED are the main entry to the hospital for AHF, with 64% of these admissions being subsequent to an ED visit.^4^ This syndrome is reportedly associated with poor outcomes, with a 80% rate of hospital admission, a median length of hospital stay of 10 days and a mortality around 10% at 30 days, and a readmission rate of 25-30% at 30 days.^5–7^

Despite a high rate of morbidity and mortality, the management of AHF has not changed for several decades and most clinical studies failed to demonstrate a positive impact of new drugs on patients’ prognosis.^8,9^European guidelines include the use of diuretics, nitrates, oxygen and non-invasive ventilation (NIV) when indicated along with the treatment of any potential AHF triggers (precipitating factors).However, these guidelines are based on moderate levels of evidence (IB and IIaB), and high-quality randomized controlled trials data (RCT) are lacking.^10–12^

In 1998 and 2000, the two cornerstones trials of Cotter et al. provided evidences of benefits associated with early vasodilatator therapy with nitrates, although on a very small sample of patients (less than 200 in total).^13,14^ Since then, every prospective trial on AHF management failed to report a clinical significant improvement of outcomes. Equipoise remains on many questions regarding the recommended therapeutics: the optimum dose and route of administration of diuretics are not clear, the use of nitrates is also debated, and the benefit of non-invasive ventilation (NIV) is unclear.^15–17^

Despite these controversies, recommendations and guidelines are published by international society (ESC, AHA, …) and constitute the basis of our understanding and standard of care.^10,11^ However, a large proportion of elderly patients in AHF do not receive adequate care, including low rate (30-50%) of nitrates therapy.^7,18,19^

In preparation of this grant application, we conducted a preliminary analysis in 8 French EDs participating in the present ELISABETH trial. For a 7-day period, we evaluated all consecutive patients aged 75 years and older with a diagnosis of AHF in the ED. Among the 73 consecutive AHF patients, 23 patients (32%) had not been investigated for the findings of precipitating factors of AHF (namely infection, acute coronary syndrome or atrial fibrillation). Moreover, among the 50 other patients for whom precipitating factors of AHF were investigated, 17 (one third) showed evidence of precipitating factors that were not subsequently treated in the ED, although diagnosed. Regarding nitrates therapy, only 25 out of the 73 included patients received recommended treatment. In total, only 18 elderly ED patients (23%) were managed according to the existing guidelines (Freund et al. 2017 manuscript under preparation).

The lack of solid evidence regarding the efficacy of full recommended therapeutic management of AHFS on outcomes may have been caused by four major shortcomings that we will address in the present ELISABETH trial:

1) The previous RCTs did not include in their protocol of care the systematic early assessment for precipitating factors, and subsequent treatment.^8,15,20,21^ The main reported triggers are acute coronary syndrome (ACS), infection, and atrial fibrillation.^22,23^ As the outcomes of AHF patient has been linked with the triggering factor, we make the hypothesis that early and comprehensive look up and treatment of these precipitating factors may improve the prognosis.^24^ A secondary analysis of the Arrigo et al. study showed that among the 15% of AHF patients with ACS, the administration of antiplatelet was associated with decreased mortality (11.4% vs 16.7%).^24^ Similarly, it is well established that the early introduction of antibiotics improve prognosis of infected patients.^25^ Along with administration of recommended treatment within the first hours of ED care, our trial will evaluate the impact of an algorithm for early detection and treatment of precipitating factors that may have contributed to the decompensation of the patient.

2) The previous RCTs only assessed the impact of single drugs, and not of a comprehensive care bundle.^8,9,15,20^ Due to polyfactorial causes of poor outcomes in elderly patients with AHF, we believe that an intervention that focuses only on the administration of a single drug may have a lesser effect than a care bundle. In the light of the high rate of sub-optimal care provided to elderly AHF patients, the inclusion of all aspect of the treatment seems mandatory to evaluate a therapeutic approach. To our knowledge, an intervention combining an early and multifaceted approach for AHF in the ED has never been studied.

3) The delay between ED arrival and randomization may have been too long: in recent large RCTs, this timeframe varied from 6 to > 24 hours.^8,20^ It has however been suggested that the introduction of decongestion treatment within hours in the ED is associated with better outcomes.^26,27^ Furthermore, a recent prospective study reported that early decongestion treatment with diuretics in the first hour was an independent predictor of improved in-hospital survival (Odds Ratio OR 0.39, [0.20-0.76]) ^28^.

In the present study, nitrates and loop diuretics will be given within 1 hours of first medical contact in the ED.

4) Although elderly are described to most suffer of AHF, with worse outcomes, specific RCT in this frail population are lacking (ex. the recent True-AHF RCT, which evaluated the effect of ularitide infusion, excluded elderly patients).^8^ Thus our trial, focused on old AHFS patients, will be to the best of our knowledge the first to evaluate the impact of an early intensive approach in this target population.

## Hypotheses for the research

In elderly patients (≥75 years), acute heart failure (AHF) is associated with a high 30-day mortality and readmission rate. We hypothesis that a care bundle, which comprises very early and aggressive decongestion treatment along with assessment and treatment of precipitating factors will improve early outcomes for elderly patients that visits the ED with AHF.

## Description of the population of research participants and justification for the choice of participants

Patients with AHF, aged 75 years and older, admitted to the ED.
We chose to focus on elderly patients for several reasons:

- These patients have higher mortality rates due to frequent co-existing illness and comorbidities.^3,4^
- Elderly patients are quite an homogenous population. They share a similarity in AHF profile, with a predominant proportion of patients with preserved ejection fraction, and similar profiles of triggers (infection in most of the cases). By contrast to younger patients (< 75 years) in AHF, who are often patients with poor left ventricular ejection fraction and require a different care pathway, mostly based on decongestion treatment, especially diuretics.^5,29^
- Elderly patients are under-represented in clinical trials, and scientific evidence is often lacking, the diagnosis and management of AHF in this population is challenging.
- Elderly patients have been extensively shown to be undertreated, whilst this population would benefit as well as patients under 75.

## Interventions and products which will be performed or used as standard

No specific drug or medical disposal will be investigated *per se*. The present trial will evaluate the efficacy of administration of full optimaltherapy (being furosemide, isosorbidedinitrate, LMWH, antibiotics, antiplatelets…) as recommended by current guidelines See section 6 below.

## Interventions added for the research

The intervention will not test any particular drug or medical disposal, but rather the application of the international guidelines (ESC, EUSEM, AHA) through the implementation of a protocol of care. The care bundle of early care with a checklist that includes the following checklist:

- Detection and treatment of AHF precipitating factors (infection, acute coronary syndrome or atrial fibrillation)

Treatment of congestion, including a protocol of nitrates titration, and low dose furosemide, as recommended by international guidelines

- Non-invasive ventilation if indicated, as recommended by international guidelines
- Preventive low molecular weight heparin, as recommended by international guidelines

## Summary of the known and foreseeable benefits and risks for the participants

Despite a small improvement in outcomes of elderly admitted for AHF within the past decades, its morbidity and mortality remains severe with a 10% rate of 30-day mortality, and 25-30% of early readmission rate.^6,7,18,29^ In the majority of cases, treatment can be initiated in the ED. However, many studies showed that a majority of these patients are still not getting recommended therapies in the ED – either for AHF *per se*, or for precipitating factors of AHF, especially acute coronary syndrome or infection.^7,16,18^

In this context, there is an urgent need of multidisciplinary management program of patients with AHF in the ED and following ED to ensure better results and adherence.^30^The great outcome improvement provided by early treatment in the ED have long been established in other settings (e.g., sepsis, myocardial infarction). Unfortunately, AHF has not been considered with this regard until recently. Some reports suggest the importance of time to introduce therapy in AHF. Data derived from ADHERE registry indicate that early treatment (<6h) in emergency departments would bear a positive impact by decreasing in-hospital mortality and morbidity rates (unadjusted OR for in-hospital mortality 0.77, adjusted OR 0.87 (95%CI [0.76 – 0.96]).^26^ Very recently, in their large prospective observational study, Matsue et al. reported a significant decreased mortality of AHF following the initiation of decongestion therapy within 1 hour in the ED (OR for in-hospital mortality of 0.39 (IC 95% [0.20-0.76]).^28^

As expressed by Januzzi and Felker in a recent editorial “***the failure of novel therapies for AHF requires us to make better use of what we already have. A systematic approach would allow an optimal management of acute HF, and in turn could finally improve outcomes.”***^31^If our hypothesis is confirmed, our trial of early intensive care bundle will be the first RCT showing a significant reduction in short term morbidity and mortality in elderly AHF, similarly to what was achieved for sepsis (with a 15% absolute reduction of in-hospital mortality).^32,33^

Lastly, it can be stressed that the observed high rate of deviation to the guidelines may be caused in part by their low level of evidence of these last one. A positive outcome of the execution of a care bundle based on these recommendations would increase physician’s adherence, and patients’ outcomes.

# OBJECTIVES OF THE RESEARCH

## Main objective of the research

To compare the efficacy of an early and comprehensive management strategy of AHF in elderly patients to the usual care on morbi-mortality at 30 days.

## Secondary objectives

To evaluate the effect of AHF management on early morbidity and mortality.

# Description of the research

## Primary endpoint

Our primary endpoint is:

**The number of days alive and out of hospital at 30 days after ED visit.**

This endpoint is considered as relevant by the group of experts of the European Society of Cardiology.^34^ In their consensus paper, the experts stated that although mortality should be captured, repeated hospitalizations should also be recorded. Especially in elderly patients, where the rate of readmission to the ED and rehospitalisation is elevated: up to 40% of heart failure admissions to the hospital are actually repeated admission for recurrence of symptoms within 30 days of a previous AHF event. ^3,6^

As expressed by Zannad et al.: “Despite their importance, repeat events are ignored in the majority of clinical trials, […], The ‘days alive and out of hospital’ endpoint incorporates the components of days in hospital (including days of the index hospitalization and repeat hospitalizations), days alive and not in hospital, and days dead into a single measure over a defined time frame (e.g. 30 or 60 days). This endpoint was developed to address the issue of repeat hospitalizations for all causes.” ^35–37^

This endpoint also makes it possible to obtain information which could have some benefits from an health-economic point of view.^34^ Furthermore, this endpoint was reported to better capture the burden of mortality and hospital stay during the follow up period. As expressed by Allen et al., this endpoint has the advantage to combine mortality, length of stay, and burden of subsequent hospital stay into a single endpoint, and therefore is appropriate to capture morbidity and mortality.^38^

Lastly, the timeframe of 30 days is recommended as a shorter timeframe would not catch recurrence and morbidity, and a longer timeframe would catch events that are more likely linked to chronic morbidity of the patients than to the AHF syndrome.^24,34,38^

## Secondary endpoints

- 30 day all-cause mortality
- 30 day cardio-vascular mortality
- Hospital readmission at 30 days
- Length of in hospital stay truncated at 30 days
- Changes of more than 2 fold in creatinine level from inclusionto day 30 or to discharge whichever comes first.

Creatinine will be measured at day 0 in the ED, and at discharge day or day30, whichever comes first.

# Research methodology

## Design of the study

The ELISABETH trial is designed as a stepped wedge cluster randomized trial. We decided to choose this design for the following reasons:

- As we implement a new protocol, there is a risk of contamination. An emergency physician, who would have already treated patients via the care bundle protocol, would be subsequently influenced by this trial, and could have difficulty to provide the former “standard of care”. Therefore, a randomization at the patient level or a cross-over design would induce bias through contamination. This bias was likely a reason why the recent “Guide It” trial failed to provide significant difference between control group and intervention group.^39^
- The present ELISABETH trial focus on a severe condition, in EDs that are often busy places, therefore the need for a randomization at the patient level could be an impediment to inclusion, and therefore limit our ability to recruit consecutive patients
- A cluster stepped-wedge design prevents contamination as centers will first be allocated to standard care, before implementing the intervention. Furthermore, a stepped wedge design would also prevent a potential “period effect” that could have resulted from a simple cluster before/after design

All the 15 participating centers will begin with a “control period” for 4 weeks. Then, every 2 weeks, one center will be randomized to switch to the “intervention period”. After 32 weeks, all centers will then be in the intervention group for a last 4-week period.

| **Weeks** | **1-2** | **3-4** | **5-6** | **7-8** | **9-10** | **11-12** | **13-14** | **…** | **27-28** | **29-30** | **31-32** | **33-34** | **35-36** |
| --- | --- | --- | --- | --- | --- | --- | --- | --- | --- | --- | --- | --- | --- |
| Center 1 | C | C | I+T | I | I | I | I | … | I | I | I | I | I |
| Center 2 | C | C | C | I+T | I | I | I | … | I | I | I | I | I |
| Center 3 | C | C | C | C | I+T | I | I | … | I | I | I | I | I |
| Center 4 | C | C | C | C | C | I+T | I | … | I | I | I | I | I |
| Center 5 | C | C | C | C | C | C | I+T | … | I | I | I | I | I |
| Center 6 | C | C | C | C | C | C | C | … | I | I | I | I | I |
| Center 7 | C | C | C | C | C | C | C | … | I | I | I | I | I |
| Center 8 | C | C | C | C | C | C | C | … | I | I | I | I | I |
| Center 9 | C | C | C | C | C | C | C | … | I | I | I | I | I |
| Center 10 | C | C | C | C | C | C | C | … | I | I | I | I | I |
| Center 11 | C | C | C | C | C | C | C | … | I | I | I | I | I |
| Center 12 | C | C | C | C | C | C | C | … | I+T | I | I | I | I |
| Center 13 | C | C | C | C | C | C | C | … | C | I+T | I | I | I |
| Center 14 | C | C | C | C | C | C | C | … | C | C | I+T | I | I |
| Center 15 | C | C | C | C | C | C | C | … | C | C | C | I+T | I |

*Figure 1: stepped wedge organisation and timetable of the trial*

C: Control period. I: Intervention period. T: training

**In the case where one or more centers will have to drop out of the trial, a mid-term reevaluation of the period distribution will be conducted by the steering committee in order to ensure similar repartitions of period and number of patients in each group.**

This trial is comparative: intervention period vs control period. In the intervention period, patients will be treated according to the care bundle. In the control period, patients will be treated as usual (TAU) by the emergency physicians, without the aid of a care bundle.

Due to the design of the study (stepped wedge) and the intervention, there will be no blinding of the physician and the patient.

## Number of participating sites

This is a multicenter trial, which involves 15 Emergency Departments in France.Patients will be recruited in the ED at the index visit.

## Avoiding and reducing bias

### Participant identification

The participants in this research will be identified as follows:

Site number (3 digits) - Sequential enrolment number for the site (4 digits) - surname initial - first name initial

This reference number is unique and will be used for the entire duration of the study.

### Randomization

This is a stepped wedge randomisation. All centers will be randomised for their time of intervention implementation. Randomisation will be computer generated by a biostatistician from URC-Est, independent of the study and before the study beginning.

This is an open trial.

# Procedure for the research

| **Whose consent must be obtained** | **Who informs the individuals and collects their consent** | **At what point the individuals are informed** | **At what point the consent is obtained** |
| --- | --- | --- | --- |
| - *the subject participating in the trial;* - orthe trustworthy person / family member / close relative *if the condition of the patient does not permit;* | - *one of the local investigators* ***(emergency physician)***   *Principal investigator or a declared collaborating doctor who has been trained in the research* ***(emergency physician)*** | - *at inclusion in the ED* - *as soon as his condition permits* | - *At inclusion D0 or as soon as his condition permits* - *before hospital discharge* |

## Schedule for the study

- inclusion period: 36 weeks
- participation period (treatment + follow-up): 30 days
- total duration: 36 weeks and 30 days

### D0 : inclusion and baseline visit

The inclusion visit takes place at day 0, during the ED index visit. Patients should be included during the first 6 hours after ED entrance.

As this is a cluster study, and the intervention consists of an application of recommended guidelines, all patients with AHF will be treated as per the care bundle according to the strategy period, whether in the study or not but their data will not be collected if patients are not included.

1. Inclusion

After having checked inclusion and exclusion criteria, the investigator will seek consent of participation.

-if the patient is able to provide his/her written informed consent : the investigator will inform the patient and obtain his/her written informed consent (for the utilization of his hospital data and 30 days follow-up).

- if the patient is unable to provide his written informed consent : the investigator will inform and obtain the consent of the trustworthy person, family member or close relative, as appropriate (Article L1122-2 of the French Public Health Code).

- if the patient is unable to provide his written informed consent and in the absence of the trustworthy person, next of kin or close relative : a procedure for inclusion in the study in emergency situation will be applied (article L1122-1-2 of the French Public Health Code). In this case, continuation-of-care consent for the study will be signed by the patient (if need be by trustworthy person, family member or close relative) as soon as possible, according to French Law (article L1122-1-2 of the French Public Health Code).

2. Baseline data

The local investigator will also collect with the help of clinical research technician the following variables at the time of the ED stay:

- Past medical history (Chronic heart failure, acute coronary syndrome, Chronic respiratory failure )
- Chronic oral intake of diuretics, nitrates, antibiotic, antiplatelet, anticoagulant.
- Baseline characteristics: heart rate, systolic and diastolic blood pressure, temperature
- ECG rhythm and signs of ischemia
- Main biological parameters if performed: haemoglobin, White blood cell count, troponin, BNP or nt-proBNP, creatinine, CRP, procalcitonin, arterial blood gas
- Treatment given in the ED in the first 4 hours:
  - Diuretics (dose and class)
  - Nitrates (dose and class)
  - Antibiotics
  - Antiplatelet or anticoagulant
  - Anti-arythmic (digoxine, cordarone)
  - Anticoagulant / LMWH
  - NIV
- Discharge disposition: home, Clinical decision unit, admission to the hospital, cardiology, admission to intensive care unit, death.

### Follow-up visits (if discharge before 30 days)

If the patient is discharged before 30 days, the last value of creatinine measurement before discharge will be collected.

### Research end date (D30)

The end of study visit will be at 30 day. The in-hospital mortality will be truncated at 30 days.

- If the patient is still hospitalized: the follow-up visit will consist of a hospital visit and a chart review to ascertain the primary endpoint.
- If the patient is not still hospitalized: the follow-up visit will consist of a phone interview topatient/ trustworthy person / family member / close relative. The family practitioner of the patients will be sought for information in case the patients/relatives cannot be reached.

If follow up is impossible, the investigators or CRT will contact the city hall and administrative service of his hometown to seek for possible death.

## Expected length of participation, chronology and duration of the study

The maximum duration between ED arrival and enrolment will be 6 hours.

After inclusion; the care bundle (treatment period) should be completed within 4 hours.

In both periods, each subject will participate in the study for 30 days (see above).

As shown in figure 1, there will be a first period of 4 weeks where all centres will be in the control arm, then every 2 weeks one centre will change arm and switch to the “intervention” phase. After a total of 32 weeks, all centers will be in the “intervention” arm, for four more weeks. The total duration of recruitment will therefore be 36 weeks.
Since patients will be followed until 30 days, the total duration of the study will then be of 36 weeks and 30 days.

|  |  |  |
| --- | --- | --- |
| Maximum period between D0 and enrolment  Length of Inclusion period |  | 6 hours  36 weeks |
| Duration of participation for each subject, of which: |  |  |
| - protocol period: |  | 4 hours |
| - Follow-up period: |  | 30 days |
| Total study duration: |  | 36 weeks and 30 days |

## Table or diagram summarizing the chronology of the research

| *Actions* | *D0 in the ED*  *(Inclusion and Baseline visit)* | *Discharge if before D30* | *D30* |
| --- | --- | --- | --- |
| *Inclusion and exclusion criteria verification* | *R* |  |  |
| *Information: (patient or trustworthy person, family member or a close relative)* | *R* | *R*  *(if emergency situation at D0)* |  |
| *Signature of the consent form (patient or* trustworthy person, family member or a close relative) | *R* | *R*** |  |
| *Past medical history* | *C* |  |  |
| *Clinical exam (auscultation and examination)* | *C* |  |  |
| *Biological test (cf 5.2)* | *C* | *C* | *(C)** |
| *ECG +/- echocardiography* | *C* |  |  |
| *Chest X-Ray* | *C* |  |  |
| *Care bundle / Treatment as usual* | *C* |  |  |
| *ED Discharge disposition* | *C* |  |  |
| *Status (in-hospital, home, dead)* |  |  | *R* |
|  |  |  |  |

** only if not done before, i.e. if still admitted in the hospital at D30 subsequent to ED visit.*

*** if not done at D0 (patient in emergency situation)*

*R: performed for research/ C: performed in the context of care*

## Distinction between standard care and research

This is an intervention study, where the intervention comprises the application of recommendations and guidelines for the management of AHF, ACS and infection. Therefore, only conventional and recommended treatments will be delivered to patients.

**Control period: Acute heart failure standard therapy:**

- Treatments are given at the discretion of the treating emergency physician

The guidelines and standard of care will be recalled to the emergency physicians at the begining of the trial in each center when the control period will start.

**Intervention period: Early intensive care bundle:** The care bundle comprises a list of items to follow and tick on a handover checklist (see below-Figure 2) within 4 hours of ED management:

- a) Treatment of the congestion: *(international guidelines and recommendations* ^10,11^)
  - 40mg of Intravenous (iv) furosemide (or usual daily dose) if not already given pre-hospital.
  - IV nitrates given in boluses of 3mg every 5 minutes. After one hour of bolus titration, then continuous infusion with an hourly dose of at least half of total given during the first hour of nitrate. Blood Pressure (BP) will be monitored every 5 minutes during the titration (then hourly), and nitrates will be discontinued if BP drops <100mmHg.
- b) Treatment of precipitating factors :
  - Administration of antibiotic therapy (accordingly to local guidelines amoxicillin and clavulanic acid in most cases) if at least two of the following: Fever > 38°C, leucocytes > 12 000 G/L, radiological signs suggestive of lower respiratory tract infection or elevated CRP or PCT,
  - Administration of dual antiplatelet therapy and transfer to cardiac intensive care unit if at least 2 of the followings: chest pain, ischemic signs on ECG, elevated troponin concentration or change in troponin concentration. These patients will be transferred for coronary angiography if indicated by the cardiologist, as recommended.^40^
  - In case of atrial fibrillation: administration of heparin, heart rate control strategy (digoxin or amiodarone as indicated) to reduce heart rate under 100 bpm, early admission to a cardiac intensive care unit if elevated troponin associated.
- c) NIV if respiratory distress with hypercapnia and pH < 7.35 in absence of contra indication.^11^
- d) Preventive LMWH if no pre-existing anticoagulation therapy.^11^

**All treatment will be initiated in the ED, and their continuation or discontinuation will be evaluated by the treating physician during the subsequent hospital stay.**

**
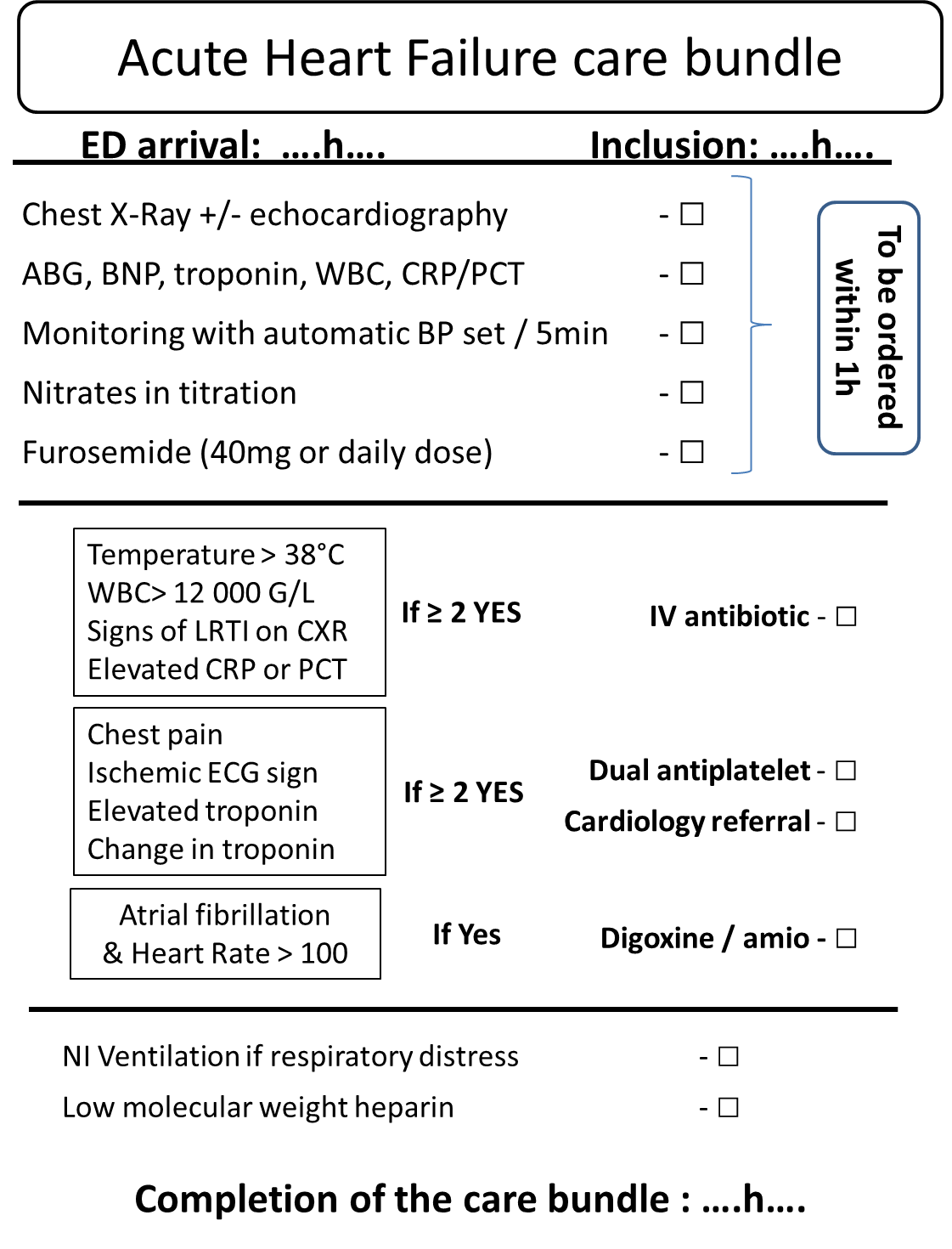
**

*Figure 2: example of handover sheets with checklist for AHF management*

During the intervention period, the clinical research technician, nurse, or investigator will have to fill the handover of treatment list, as reproduced below:


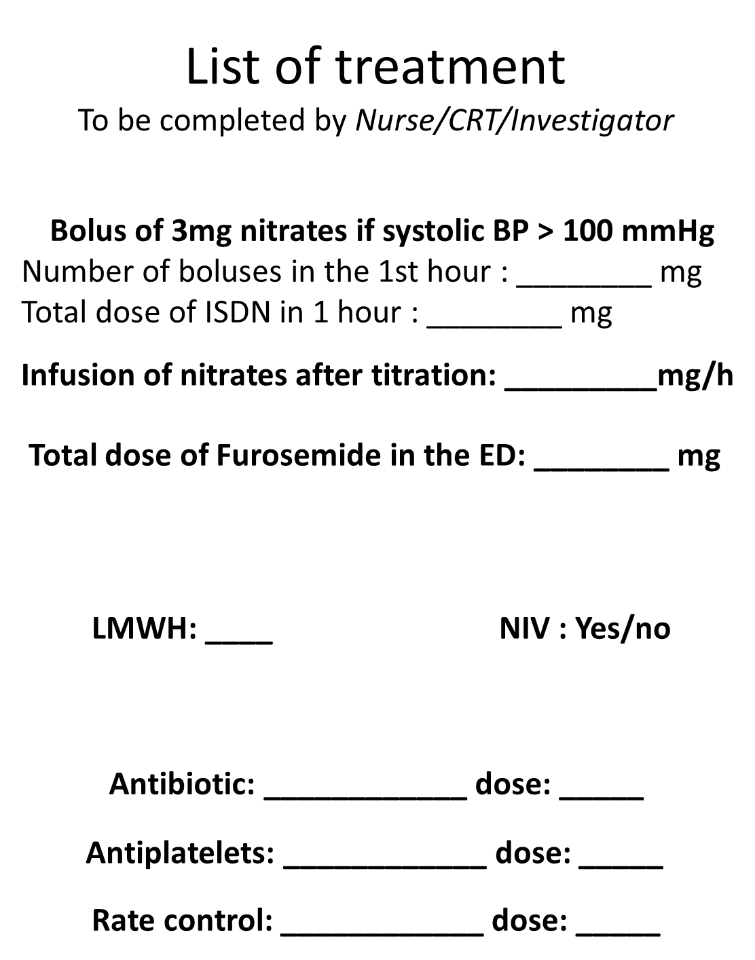


*Figure 3: list of treatment received in the ED*

## Biological samples

No biological sample will be stored during the trial.

# ELIGIBILITY CRITERIA

## Inclusion criteria

- Age ≥ 75 years
- Admission to the emergency departmentwith a diagnosis of AHF determined by the emergency physician, based on the presence of:
  - at least one of the following symptoms: acute, or worsening of dyspnea, orthopnea
  - one or more of the following: pulmonary rales, peripheral edema, a chest radiograph or transthoracic ultrasound showing pulmonary vascular congestion signs, increased natriuretic peptides (BNP or NT pro BNP).
  - Patients affiliated to French social security (“AME excepted”))
  - Written informed consent signed by the patient / the trustworthy person / family member / close relative, or inclusion in case of emergency and written informed consent will been signed by the patient (if need be by trustworthy person, family member or close relative) as soon as possible (article L1122-1-2 of the French Public Health Code)

## Exclusion criteria

Patients are excluded if they have any of the followings:

- Other obvious cause of acute illness (severe sepsis, ST elevation Myocardial infarction)
- Systolic blood pressure less than 100mmHg
- Any contra-indication to nitrates (severe mitral or aortic stenosis, or severe aortic regurgitation)
- Known chronic kidney injury on dialysis
- Time from ED entrance to inclusion > 6h,
- Patient under legal protection measure (tutorship or curatorship) and patient deprived of freedom

## Enrolment procedure

The 15 recruiting centers are:

| **City** | **Country** | **Hospital** | **Unit** |
| --- | --- | --- | --- |
| Paris | France | Pitié-Salpêtrière | Service des Urgences |
| Bobigny | France | Avicenne | Samu 93 |
| Paris | France | Saint-Antoine | Service d’Accueil des Urgences |
| Paris | France | HEGP | Service d’Accueil des Urgences |
| Tours | France | CHU Tours | Département de Médecine d’Urgences |
| Nîmes | France | CHU Nîmes | Pôle Anesthésie Réanimation Douleurs Urgences |
| Nancy | France | CHU Nancy | Service des Urgences / SAMU / SMUR |
| Toulouse | France | CHU Rangueuil | Service des Urgences |
| Paris | France | Lariboisiere | Service des Urgences / SMUR |
| Paris | France | Cochin | Service des Urgences |
| Nice | France | CHU Nice | Service des Urgences |
| Creteil | France | CHU Henri Mondor | Service des Urgences |
| Boulogne | France | CHU Ambroise Paré | Service d’Accueil des Urgences |
| Paris | France | CH Saint-Joseph | Service des Urgences et Lits d’Urgence |
| Besançon | France | CHU Besancon | Service d’Accueil des Urgences |

In the case where one center withdraw from the trial before its start, we will invite another ED to participate (two centers are listed as willing to participate if possible – CHU Nantes and CHU Lyon).

Patients will be included by the emergency physicians whilst working in the ED. After a patient is suspected of AHF, the physician in charge (with the help of clinical research technician if present) will sought any exclusion criteria before seeking patient’s informed consent. To improve adherence to the bundle, after each center switches to the intervention period, a clinical research technician will be working in the ED during training period).

The present research team and its study coordinator (Y Freund) has conducted several previous studies in these 15 centers (among others) where patients were included and followed as planned: 1000 patients (30 centres) in 1 month [NCT02738164, Freund et al. JAMA 2017]; 500 patients (4 centers) in 1 year [NCT01774500 PlosOne 2014]; 1800 patients (14 centres) in 1 year (NCT02375919, *JAMA 2018*); 550 patients (13 centers) in 3 months (NCT02926664,*, EJEM 2018)*). Each of these prospective multicenter studies coordinated by Y. Freund, the scientific director of the present ELISABETH trial achieved full targeted recruitment within the pre specified timeframe.

The scientific director(A Mebazaa) of the ELISABETH trial was (among other) the European chairman of the True-AHF study, a trial that recruited similar patients in some of the participating centers. The target recruitment has been completed in time (NEJM 2017). AM was also the study coordinator of a previous PHRC funded trial (Frog –ICU), which also achieved full recruitment in time *(in revision, Critical Care)*.

|  | *Number of subjects* |
| --- | --- |
| *Total number of subjects to be included* | *500* |
| *Number of sites* | *15* |
| *Enrolment period (months)* | *36 weeks* |
| *Number of subjects/site* | *33* |
| ***Number of subjects/site/month*** | ***4*** |

AHF in elderly is a common diagnosis in the ED.

In the preliminary study that assessed our potential of recruitment in 8 of the participating centers, the median number of included patients was 10 per week per center (range 6 to 13) when using the same inclusion and exclusion criteria than the present trial, the median number of included patients was 10 per week per center (range 6 to 13):

- **Nancy**: 13 patients / week
- **Besançon**: 6 patients / week
- **Pitié-Salpêtrière**: 11 patients / week
- **Saint-Antoine**: 9 patients / week
- **HEGP**: 6 patients / week
- **Tours**: 12 patients / week
- **Lariboisiere**: 11 patients / week
- **Nimes**: 6 patients / week

The remaining 7 recruiting centers that will participate in the present ELISABETH trial are EDs of similar size and settings, therefore with the same recruitment potential.

**Thus, the target of 4 patients per month per site seems highly achievable.**

*In the case where one center withdraw from the trial before its start, we will invite another ED to participate (two centers are listed as willing to participate if possible – CHU Nantesand CHU Lyon). In the case where one center should withdraw from the study after the beginning of the trial, the steering committee will evaluate mid-term recruitment after 16 weeks and adjust the remaining period to ensure that all groups will have the same total period length of inclusion, and similar sample size.*

## Inclusion rate

We aim to have balanced population between the two groups, especially in terms of number of patients analysed in each group. Furthermore, to avoid any period effect, centers must include their targeted number of patients throughout the whole inclusion period. Therefore, we will closely monitor the number of patient recruited in each center. The local investigator or the CRT will follow at least once every 2 weeks how many patients were included and could be analysed.

# Termination and exit rules

## Criteria and procedure for premature withdrawals and exits from the study

- Subjects may exit the study at any time and for any reason.
- The investigator can temporarily or permanently withdraw a subject from the study for any safety reason or if it is in the subject's best interests.

🡺Subject lost to follow-up: the subject cannot be located. The investigator must make every effort to reconnect with the subject (and record his attempts in the source file), at least to determine whether the subject is alive or dead.

If a subject exits the trial prematurely or withdraws consent, any data collected prior to the date of premature exit may still be used.

The case report form must list the various reasons why the subject exited or was withdrawn from the study:

- Subject's personal reasons
- Explicit withdrawal of consent
- Lost to follow-up

### Procedure for replacing participants

If a subject exits the trial,this will in no way affect the standard care received for his/her condition.

### Full or partial cancellation of the study

AP-HP, as the sponsor, reserves the right to permanently suspend enrolment at any time if the enrolment targets have not been met.

# EFFICACY ASSESSMENt

## Description of parameters for assessing efficacy endpoints

The primary endpoint (days alive and out of hospital at day 30) will be measured at the end of the 30 days follow up period, either by hospital visit, phone interview, and medical chart review. Vital status, date of death and date of discharge will be collected.

A death during the follow up period will correspond to 0. An ED visit will correspond to “one day” at the hospital.

For example, a patient not admitted (at day 0), with no return visit to the hospital, and alive at day 30 will have 30 days alive and out of hospital.

A patient who is admitted (at day 0) and stays 8 days in the hospital before being discharged and have no readmission and no return visit to the ED would have a “22 days alive and out of hospital at 30 days”.

A patient that is admitted and die at 13 days, either at home or in hospital will have 0.

A patient that is admitted for 10 days, discharged home for 5 days then admitted at day 16 for 15 days will have 5 days alive and out of hospital (namely day 11, 12, 13, 14 and 15).

## Anticipated methods and timetable for measuring, collecting and analyzing the efficacy data

- In-hospital mortality will be obtained from hospital data base (Mediweb, Gilda, or Orbis) and phone interview with the last known ward of hospitalization of the patient. In case of transfer to another hospital, this data will be collected either by phone interview or hospital visit if necessary.
- 30 day all-causes mortality rate: same as above for patient still in hospital at day 30, phone call to the patients, relatives, or GP if not.
- Cardiovascular related 30 day mortality. Adjudicated by an adjudication committee (see below) as to whether the death has been mainly caused by a cardiovascular issue.
- Length of stay in hospital and changes of more than 2 fold in creatinine level from hospital database (CleanWeb, MediWeb, Gilda or Orbis)
- Efficacy data will be analyzed at the end of the study after data review and freezing of data base.

# SAFETY

During this research, adverse events (serious and non-serious) do not need to be reported to the sponsor. The report must instead be made as part of the vigilance procedure applicable to the product or intervention under investigation (pharmacovigilance

for a drug product; medical device vigilance for a medical device, etc.).

# Specific committee for the study

## Steering Committee

**Members of the committee**: PrAlexandreMebazaa (Primary investigator, anaesthesiologist), Dr Yonathan Freund (scientific coordinator, emergency physician), PrSaïd Laribi (emergency physician), Pr Alain Cohen-Solal (Cardiologist), Pr Jacques Boddaert (geriatrician), PrTabassome Simon (clinical pharmacologist; and Methodologist), Marine Cachanado (statistician), a representant of the promotor (DRCI head office project advisor)

**Missions:**Define the overall structure of the study, coordinate information, review the initial methodology and oversee the trial.

The committee can be asked to adjudicate any death in the study and relate whether it is from cardiovascular cause.

Rhythm of the meeting: 1meeting per years.

# data management

## Data collection

Data will be collected in an electronic case report form (e-CRF), devised by the study coordinator in collaboration with URC-EST. Baseline data will be completed by the investigators with the help of a Clinical Research Technician (CRT) of URC-Est for AP-HP centers and a local Clinical Research Technician for other centers.

## Identification of data recorded directly in the CRF which will be considered as source data

After inclusion, the local investigator, emergency physician, with the help of the CRT if present, will complete ae-CRF that contains inclusion/exclusion criteria, main baseline characteristics and will complete the handover checklist (care bundle Figure 2).
The outstanding variables will be collected through the review of electronic medical chart of ED visit (Urqual in most centers).

Data regarding follow up, survival and hospital evolution will be collected through the review of electronic medical chart of ward hospitalization (MEdiweb), administrative software (Gilda / Orbis) or if needed physicians’/CRT phone call.

## Right to access source data and documents

### Data access

In accordance with GCP:

- the sponsor is responsible for ensuring all parties involved in the study agree to guarantee direct access to all locations where the study will be carried out, the source data, the source documents and the reports, for the purposes of the sponsor's quality control and audit procedures.

- the investigators will ensure the persons in charge of monitoring and auditing the research and of quality control have access to the documents and personal data strictly necessary for these tasks, in accordance with the statutory and regulatory provisions in force.

### Source documents

The source documents are any original document or item that proves the existence or accuracy of a data-point or fact recorded during the study. Source documents will be kept by the investigator, or by the hospital in the case of hospital medical records, for the statutory period.

That will include:

- handover checklist and treatment list
- Medical chart, laboratory test results and imaging reports from the initial (or repeated) hospitalisation

### Data protection

The persons responsible for the quality control of clinical studies (Article L.1121-3 of the Code de la Santé Publique - CSP (French Public Health Code) will take all necessary precautions to ensure the confidentiality of information relating to the research, the participants and in particular their identity and the results obtained.

These persons, as well as the investigators themselves, are bound by professional secrecy (in accordance with the conditions set out in Articles 226-13 and 226-14 of the French Criminal Code).

During and after the research, all data collected about the participants and sent to the sponsor by the investigators (or any other specialized collaborators) will be anonymized.

Under no circumstances will the names and addresses of the participants be shown.

The sponsor will ensure that each participant has agreed in writing for any personal information about him or her which is strictly necessary for the quality control of the study to be accessed.

## Data processing and storage of research documents and data

### Identification of the person responsible and the location for data processing

Data management will be performed by a data manager from URC-Est under the responsibility of Pr T. Simon. Statistical analysis will be performed by a biostatistician from URC-Est under the responsibility of Pr T. Simon (Pr T. Simon).

### Data entry

Data will be entered electronically via a web browser.

### Data processing (CNIL, the French Data Protection Authority)

This research is governed by the CNIL "Reference Method for processing personal data for clinical studies" (MR-001, amended). AP-HP, the sponsor, has signed a declaration of compliance with this "Reference Method" Adapt based on the internal procedures of the data management entity

All personal data for this research will be processed in accordance with Chapter IX of the amended French Data Protection Act of 6 January 1978 (articles 53-61).

### Archiving

All specific documents for Minimal Risk and Restriction research studies are to be archived by the investigator and the sponsor for 15 years after the end of the research.

This indexed archiving applies to:

- A sealed envelope for the investigator, containing one original of all information sheets and consent forms signed by all individuals at the site who participated in the research;
- A sealed envelope for the sponsor, containing one copy of all information sheets and consent forms signed by all individuals at the site who participated in the research;
- "Study" binders for the Investigator and the sponsor, containing (non-exhaustive list):
- all successive versions of the protocol (identified by version no. and date), and its appendices
- decisions of the CPP
- correspondence
- the enrolment list or register
- the appendices specific to the research
- Final report
- The case report forms

## Ownership of the data

AP-HP is the owner of the data. The data cannot be used or disclosed to a third party without its prior permission.

# statistical aspects

## Description of statistical methods to be used including the timetable for the planned interim analyses

No interim analysis is planned. Analysis will be performed at the end of the study after data review and freezing of data base.

Analyses will be performed using SAS® software (version 9.3 or updated version).

Principal analysis will be realized according to the ITT principle.

Baseline patient’s characteristics will be considered at both with the cluster (center) and patient level.

For the center level, characteristics at the beginning of the study will be described (there are no expected change between the two periods for cluster characteristics).

Baseline characteristics of patients will be described globally and according to the period. Continuous variables will be summarized using descriptive statistics, i.e number of subjects, mean, standard deviation (s.d), median, inter quartile range, minimum and maximum. Qualitative variables will be summarized by frequency and percentage.

Number of cross-over from one group to the other group will be described.

## Principal criterion analysis

The number of days alive and out of hospital will be calculated based on date of admission, vital status, date of death and date of discharge will be collected. A death during follow-up will correspond to 0. An ED visit will correspond to “one day” at the hospital.

The number of days alive and out of hospital will be analysed using a linear regression mixed model with a random effect for each cluster, considered fixed effects will be: strategy and, for the stepped wedge design, time representing each step. In case of non-normality distribution of the interest variable, a transformation could be realized.

## Secondary evaluation criteria

In hospital mortality, all causes mortality at 30 days and cardiovascular mortality at 30 days will be compared between groups by using Pearson's chi-square test or Fisher exact test.

If possible, generalized linear regression mixed model with Poisson distribution will be performed. If the number of events is sufficient, generalized linear regression mixed model using logit link will be performed.

The length of stay in hospital in days will be compared between the two periods by using Student t-test or Wilcoxon rank-sum test as needed. If possible, a linear regression mixed model will be performed. A random effect for each cluster will be considered and considered fixed effects will be: strategy and, for the stepped wedge design, time representing each step. In case of non-normality distribution of the interest variable, a transformation could be realized.

Percentage of patients with a change of more than 2 fold in creatinine between inclusion and 30 days will be compared between groups by using Pearson's chi-square test or Fisher exact test. If possible, generalized linear regression mixed model with Poisson distribution will be performed. If the number of events is sufficient, generalized linear regression mixed model using logit link will be performed.

Second analysis will be performed on the per protocol population.

## Calculation hypotheses for the number of subjects required and the result

From our previous cohort, the mean number of days alive and out of hospital at 30 days was 14±9. To be clinically relevant, we estimate that the new approach should increase this endpoint of 3 days at least (a relative increase of 20%). With a power of 80% and alpha=5%, we need to include 283 patients. Since this study is planned as a stepped wedge cluster and after specification of following elements: 15 clusters, ICC=0.0001, the design effect is estimated at 1.609, so we need to analyze454patients – to take into account 10% of non-evaluable patients, it is necessary to include 500 patients -around 2 per cluster for each 2 weeks period.

## Anticipated level of statistical significance

All tests will be performed at 5%.

## Statistical criteria for termination of the study

Not applicable.

## Method for taking into account missing, unused or invalid data

Missing value for the principal criteria will be considered as failure (0 days alive and out of the hospital), whatever the period considered.

Sensitivity analyses will be performed to check the impact of replacement methods of missing values with missing data considered: 1) success (maximum days alive and out of the hospital observed in the total population of the study) in the experimental group and as failure (0 days alive and out of the hospital) in the control group; 2) failure in the experimental group and as success in the control group.

Others missing data will not be replaced.

- 1. Management of modifications made to the analysis plan for the initial strategy

Modification made in analysis will be documented in the final report.

## Choice of individuals to include in the analyses

ITT population: all included patients according to the period assigned by the randomization to the center, regardless of the strategy effectively received by the patient.

Per protocol population: all included patients without major protocol deviation:

- No respect of selection criteria,
- No respect of strategy assigned by randomization (cross-over for example),
- Missing value for the principal criteria,
- Other major protocol deviation identified during data review and freezing of data base.

# QUALITY **CONTROL AND ASSURANCE**

Every clinical study managed by AP-HP is ranked according to the projected risk incurred by the study participants using a classification system specific to AP-HP-sponsored clinical trials.

## General organization

The sponsor must ensure the safety and respect of individuals who have agreed to participate in the study. The sponsor must have a quality assurance system for monitoring the implementation of the study at the research centers.

The sponsor will establish a system for opening the research centers and may also implement a data quality control system.

The sponsor shall appoint Clinical Research Associates (CRA) whose primary role is to carry out regular follow-up visits at the study sites, after completing their initial visits.

The purpose of monitoring the study, as defined in the Good Clinical Practices (GCP section 5.18.1), is to verify that:

• the research subjects are safe, protected and their rights are being met

•the data being recorded is accurate, complete and consistent with the source documents

•the study is carried out in accordance with the current version of the protocol, with GCP and with all statutory and regulatory requirements.

### Strategy for site opening

The strategy for opening the centers will be determined before the research begins.

Opening visit will be carried out by the local investigator and at least one member of the steering committee and one member of URC-Est. ED physicians will be invited to participate in the opening session in each center. A similar visit will occur at the time of the period switch, with the presence of one member of URC-Est, one member of the steering committee and the local investigator.

### Data quality control

For this study, the appropriate monitoring level has been determined based on the complexity, the impact and the budget for the study. Therefore the sponsor, in agreement with the coordinating investigator, has agreed on a logistical score and impact and the corresponding study monitoring level of: **minimal** level.

A Clinical Research Associate (CRA) appointed by the sponsor will be responsible for the proper running of the study, for collecting, documenting, recording and reporting all handwritten data, in accordance with the Standard Operating Procedures applied within the DRCI.

The investigator and the members of the investigator's team agree to make themselves available during regular Quality Control visits by the Clinical Research Associate.

During these visits, the following elements will be reviewed:

- written consent
- compliance with the study protocol and its procedures
- quality of the data collected in the case report forms: accuracy, missing data, consistency of the data with the "source" documents (medical files, appointment books, original copies of laboratory results, etc.)

## Case report forms

All information required by the protocol must be entered in the case report forms. The data must be collected as and when they are obtained, and must be written clearly and legibly. Any missing data must be coded.

Every site will have access to the electronic case report forms via a web-based data collection system. Investigators will be given instructions for using this tool.

Using on-line case report forms means the CRA can view the data quickly and remotely. The investigator is responsible for the accuracy, quality and relevance of all the data. In addition, there are consistency checks to ensure the data are verified immediately upon being entered. The investigator must validate any changes to the values in the case report form. An audit trail will be kept of all changes. A justification can be added when applicable, as a comment.

A print-out, authenticated (signed and dated) by the investigator, will be requested at the end of the research. The sponsor will keep the original. The investigator must keep a copy.

## Management of non-compliances

Any events that occur as a result the investigator or any other individual involved in conducting the study failing to comply with the protocol, standard operating procedures or statutory and regulatory requirements must be recorded in a declaration of non-compliance and sent to the sponsor.

The sponsor has its own procedures for managing these non-compliances.

## Audits

The investigators agree to accept the quality assurance audits carried out by the sponsor as well as the inspections carried out by the competent authorities. All data, documents and reports may be subject to regulatory audits. These audits and inspections cannot be refused on the grounds of medical secrecy.

An audit can be carried out at any time by [independent individuals appointed by the sponsor.](http://www.chusa.jussieu.fr/urcest/sous_cadre.php?fich=Lexique/new_index.php?isphp=0&fich=EC/legislation/DispositionslegislativesPromoteur.htm) The aim of the audits is to ensure the quality of the study, the validity of the results and compliance with the legislation and regulations in force.

The persons who manage and monitor the study agree to comply with the sponsor's audit requirements.

The audit may encompass all stages of the study, from the development of the protocol to the publication of the results and the storage of the data used or produced as part of the study.

## Principal Investigator's declaration of responsibility

Before starting the study, each investigator will give the sponsor’s representative a signed and dated copy of his/her most recent curriculum vitæ, produced within the past year, and RPPS number (RépertoirePartagé des Professionnels de Santé, Collective Database of Health Professionals). The CV must describe any previous participation in clinical research and related training.

Each investigator will agree to comply with legislation and to conduct the study in line with regulations, in accordance with the Declaration of Helsinki.

The Principal Investigator at each participating site will sign a declaration of responsibility (standard DRCI document) which will be sent to the sponsor's representative.

The investigators and their co-workers will sign a delegation form specifying each person's role and must supply their CV.

# ETHICAL AND LEGAL CONSIDERATIONS

## Methods for informing and obtaining consent from the research participants

*This is a cluster stepped wedge randomised trial, in other words randomization is performed at the hospital level and concerns the switch from control period (no intervention in care) to interventional period (focus on standard care recommendations). The informed consent of the patients will be required to collect his in-hospital data in the eCRF, and the 30-day follow up.*

In accordance with Article L.1122-1-1 of the Code de la Santé Publique - CSP (French Public Health Code), no Minimal Risk and ***Constraints*** research can be carried out on a person without his/her free and informed consent, obtained expressly after the person has been given the information specified in Article L.1122-1 of said Code.

The person will be given a reflection period between receiving the information and being asked to sign the consent form.

**If the patient is alert and considered capable of giving consent by the physician in charge:**

After screening, the emergency physician will inform the patient on the aim and details of the study.

In accordance with Article L.1122-1-1 of the French Public Health Code, no research can be carried out on a person without his/her free and informed consent, obtained in writing after the person has been given the information specified in Article L.1122-1 of said Code.

In addition, the investigator will specify in the research participant’s medical file the methods used for obtaining their consent [*or the consent of any other person, in the cases described in Articles L.1122-1-1 to L.1122-2 CSP*] as well as the methods used for providing information with a view to obtaining consent. The investigator will retain the original signed and dated consent form The information sheet and a copy of the consent form, signed and dated by patient and by the investigator or the doctor representing the investigator, will be given to the individual.

**If the person is unable to give his or her written consent:**

The consent may be obtained, in descending order of priority, from a legal representative, family member or a close relative. These persons must have no connection whatsoever to the investigator or the sponsor.

In accordance with Article L.1122-1-3, since this trial focus on an emergency condition with high mortality and morbidity, the signature of the consent by the patient can be obtained after the treatment has been started. The patient’s free and informed written consent will be obtained as soon as the clinical condition of the patient allow it by the investigator, or by a doctor representing the investigator.

if the patient is unable to provide his written informed consent and in the absence of the trustworthy person, next of kin or close relative : a procedure for inclusion in the study in emergency situation will be applied (article L1122-1-2 of the French Public Health Code). In this case, continuation-of-care consent for the study will be signed by the patient (if need be by trustworthy person, family member or close relative) as soon as possible, according to French Law (article L1122-1-2 of the French Public Health Code).

**If the patient is a protected adult,** in accordance with L.1122-2, the consent will be sought from his legal guardian.Furthermore, in case of death of the patient included in emergency situation, data collected will be used and conserved as part of research.

## Prohibition of concomitant clinical studies participation and exclusion period after the study, if applicable

Whilst participating in this trial, subjects may not take part in any other clinical study without first speaking to the doctor in charge of this trial. A participation in a non-interventionnal, observational study can be allowed during the trial. There is no exclusion period after the participation of the subject.

## Compensation for participants

### Reimbursement of out-of-pocket expenses

Not Applicable

### Compensation

Not Applicable

## Registration on a national register of clinical research participants

Not Applicable

## Legal obligations

Assistance PubliqueHôpitaux de Paris (AP-HP) is the sponsor of this study and has delegated powers to its Clinical Research and Innovation Department (DRCI) in order to conduct the study in accordance with Article L.1121-1 of the Code de la Santé Publique - CSP (French Public Health Code).AP-HP reserves the right to terminate the study at any time for medical or administrative reasons. In this case, the investigator will be informed accordingly.

## Request for approval from the CPP

AP-HP, as sponsor, obtains prior approval from the CPP for its Minimal Risk and Restriction research studies, within the scope of the Board's authority and in accordance with statutory and regulatory requirements.

## Informing the ANSM

The AP-HP will send the approval from the CPP and the summary of the protocol to the ANSM, for information.

## Declaration of compliance with the MR 001 "Reference Method"

AP-HP, the study sponsor, has signed a declaration of compliance with this "Reference Method".

## Modifications to the study

Any substantial amendment made to the protocol must be sent to the sponsor for approval. Once approval has been received from the sponsor, it must also obtain approval from the CPP before the amendment can be implemented.

The information sheet and the consent form can be revised if necessary, in particular if there is substantial amendment to the study or if adverse reactions occur.

- 1. **Final Study report**

The final study report referred to in CSP Article R.1123-67 is written and signed by the sponsor and the investigator. A report summary, meeting the competent authority’s guidelines, has to be sent to the competent authority and Institutional Review Board within one year of the end of the trial i.e. the end of the participation of the last study participant.

# Funding and Insurance

## Sources of monetary support

Programme Hospitalier de Recherche Clinique- PHRC 2017 (Ministère de la Santé)

## Insurance

For the duration of the study, the Sponsor will take out an insurance policy covering the sponsor’s own third party liability as well as the third party liability of all the doctors involved in the study. The sponsor will also provide full compensation for any damages caused by the study to the study participants and their beneficiaries, unless the sponsor can prove that the harm is not the fault of the sponsor or any agent. Compensation cannot be refused on the grounds of a third party act or the voluntary withdrawal of the person who initially consented to participate in the study.

Assistance Publique-Hôpitaux de Paris (AP-HP) has taken out insurance with HDI-GERLING through BIOMEDIC-INSURE, covering its own third party liability and that of any agent (doctor or research staff), in accordance with Article L.1121-10 of the Code de la Santé Publique - CSP (French Public Health Code).

# Publication rules

## Mention of AP-HP affiliation for projects sponsored by AP-HP

- If an author has several affiliations, the order in which the institutions are mentioned (AP-HP, University, INSERM, etc.) is not important

- However, if the research is funded by an internal call for tenders at the AP-HP, the first affiliation must be "AP-HP"

- Each of these affiliations must be identified by an address and separated by a semicolon

- The AP-HP institution must feature under the acronym “AP-HP” first in the address, specifically followed by: AP-HP, hospital, department, city, postcode, France

## Mention of the sponsor AP-HP (DRCI) in the acknowledgements of the text

“The sponsor was Assistance Publique – Hôpitaux de Paris (Clinical Research and Innovation Department)"

## Mention of the funder in the acknowledgements of the text

The study was funded by a grant from Programme Hospitalier de Recherche Clinique - PHRC 2017 (Ministry of Health)”

This research program will be registered on the website http://clinicaltrials.gov/ (include the registration number once registered).

# References

1. Arrigo M, Parissis JT, Akiyama E, Mebazaa A. Understanding acute heart failure: pathophysiology and diagnosis. *Eur Heart J Suppl*. 2016;18(suppl_G):G11-G18. doi:10.1093/eurheartj/suw044.

2. Dickstein K, Cohen-Solal A, Filippatos G, et al. ESC guidelines for the diagnosis and treatment of acute and chronic heart failure 2008: the Task Force for the diagnosis and treatment of acute and chronic heart failure 2008 of the European Society of Cardiology. Developed in collaboration with the Heart Failure Association of the ESC (HFA) and endorsed by the European Society of Intensive Care Medicine (ESICM). *Eur J Heart Fail*. 2008;10(10):933-989. doi:10.1016/j.ejheart.2008.08.005.

3. Ezekowitz JA, Bakal JA, Kaul P, Westerhout CM, Armstrong PW. Acute heart failure in the emergency department: short and long-term outcomes of elderly patients with heart failure. *Eur J Heart Fail*. 2008;10(3):308-314. doi:10.1016/j.ejheart.2008.01.014.

4. Logeart D, Isnard R, Resche-Rigon M, et al. Current aspects of the spectrum of acute heart failure syndromes in a real-life setting: the OFICA study. *Eur J Heart Fail*. 2013;15(4):465-476. doi:10.1093/eurjhf/hfs189.

5. Teixeira A, Parenica J, Park JJ, et al. Clinical presentation and outcome by age categories in acute heart failure: results from an international observational cohort. *Eur J Heart Fail*. September 2015. doi:10.1002/ejhf.330.

6. Storrow AB, Jenkins CA, Self WH, et al. The burden of acute heart failure on U.S. emergency departments. *JACC Heart Fail*. 2014;2(3):269-277. doi:10.1016/j.jchf.2014.01.006.

7. Freund Y, Delerme S, Boddaert J, Baker E, Riou B, Ray P. Isosorbide dinitrate bolus for heart failure in elderly emergency patients: a retrospective study. *Eur J Emerg Med Off J Eur Soc Emerg Med*. 2011;18(5):272-275. doi:10.1097/MEJ.0b013e328345d72a.

8. Packer M, O’Connor C, McMurray JJV, et al. Effect of Ularitide on Cardiovascular Mortality in Acute Heart Failure. *N Engl J Med*. 2017;376(20):1956-1964. doi:10.1056/NEJMoa1601895.

9. Cannon JA, McKean AR, Jhund PS, McMurray JJV. What can we learn from RELAX-AHF compared to previous AHF trials and what does the future hold? *Open Heart*. 2015;2(1):e000283. doi:10.1136/openhrt-2015-000283.

10. McMurray JJV, Adamopoulos S, Anker SD, et al. ESC Guidelines for the diagnosis and treatment of acute and chronic heart failure 2012: The Task Force for the Diagnosis and Treatment of Acute and Chronic Heart Failure 2012 of the European Society of Cardiology. Developed in collaboration with the Heart Failure Association (HFA) of the ESC. *Eur Heart J*. 2012;33(14):1787-1847. doi:10.1093/eurheartj/ehs104.

11. Mebazaa A, Yilmaz MB, Levy P, et al. Recommendations on pre-hospital & early hospital management of acute heart failure: a consensus paper from the Heart Failure Association of the European Society of Cardiology, the European Society of Emergency Medicine and the Society of Academic Emergency Medicine. *Eur J Heart Fail*. 2015;17(6):544-558. doi:10.1002/ejhf.289.

12. Collins SP, Storrow AB, Levy PD, et al. Early management of patients with acute heart failure: state of the art and future directions--a consensus document from the SAEM/HFSA acute heart failure working group. *Acad Emerg Med Off J Soc Acad Emerg Med*. 2015;22(1):94-112. doi:10.1111/acem.12538.

13. Cotter G, Metzkor E, Kaluski E, et al. Randomised trial of high-dose isosorbide dinitrate plus low-dose furosemide versus high-dose furosemide plus low-dose isosorbide dinitrate in severe pulmonary oedema. *Lancet*. 1998;351(9100):389-393. doi:10.1016/S0140-6736(97)08417-1.

14. Sharon A, Shpirer I, Kaluski E, et al. High-dose intravenous isosorbide-dinitrate is safer and better than Bi-PAP ventilation combined with conventional treatment for severe pulmonary edema. *J Am Coll Cardiol*. 2000;36(3):832-837.

15. Felker GM, Lee KL, Bull DA, et al. Diuretic Strategies in Patients with Acute Decompensated Heart Failure. *N Engl J Med*. 2011;364(9):797-805. doi:10.1056/NEJMoa1005419.

16. Mebazaa A, Longrois D, Metra M, et al. Agents with vasodilator properties in acute heart failure: how to design successful trials. *Eur J Heart Fail*. 2015;17(7):652-664. doi:10.1002/ejhf.294.

17. Gray A, Goodacre S, Newby DE, et al. Noninvasive ventilation in acute cardiogenic pulmonary edema. *N Engl J Med*. 2008;359(2):142-151. doi:10.1056/NEJMoa0707992.

18. Lemachatti N, Philippon A-L, Bloom B, et al. Temporal trends in nitrate utilization for acute heart failure in elderly emergency patients: A single-centre observational study. *Arch Cardiovasc Dis*. 2016;109(8-9):449-456. doi:10.1016/j.acvd.2016.01.014.

19. Mebazaa A, Parissis J, Porcher R, et al. Short-term survival by treatment among patients hospitalized with acute heart failure: the global ALARM-HF registry using propensity scoring methods. *Intensive Care Med*. 2011;37(2):290-301. doi:10.1007/s00134-010-2073-4.

20. Teerlink JR, Cotter G, Davison BA, et al. Serelaxin, recombinant human relaxin-2, for treatment of acute heart failure (RELAX-AHF): a randomised, placebo-controlled trial. *Lancet Lond Engl*. 2013;381(9860):29-39. doi:10.1016/S0140-6736(12)61855-8.

21. Cuffe MS, Califf RM, Adams KF, et al. Short-term intravenous milrinone for acute exacerbation of chronic heart failure: a randomized controlled trial. *JAMA*. 2002;287(12):1541-1547.

22. Diaz A, Ciocchini C, Esperatti M, Becerra A, Mainardi S, Farah A. Precipitating factors leading to decompensation of chronic heart failure in the elderly patient in South-American community hospital. *J Geriatr Cardiol JGC*. 2011;8(1):12-14. doi:10.3724/SP.J.1263.2011.00012.

23. Berkovitch A, Maor E, Sabbag A, et al. Precipitating Factors for Acute Heart Failure Hospitalization and Long-Term Survival. *Medicine (Baltimore)*. 2015;94(52). doi:10.1097/MD.0000000000002330.

24. Arrigo M, Tolppanen H, Sadoune M, et al. Effect of precipitating factors of acute heart failure on readmission and long-term mortality. *ESC Heart Fail*. 2016;3(2):115-121. doi:10.1002/ehf2.12083.

25. Seymour CW, Gesten F, Prescott HC, et al. Time to Treatment and Mortality during Mandated Emergency Care for Sepsis. *N Engl J Med*. 2017;376(23):2235-2244. doi:10.1056/NEJMoa1703058.

26. Peacock WF, Emerman C, Costanzo MR, Diercks DB, Lopatin M, Fonarow GC. Early vasoactive drugs improve heart failure outcomes. *Congest Heart Fail Greenwich Conn*. 2009;15(6):256-264. doi:10.1111/j.1751-7133.2009.00112.x.

27. Mebazaa A, Pang PS, Tavares M, et al. The impact of early standard therapy on dyspnoea in patients with acute heart failure: the URGENT-dyspnoea study. *Eur Heart J*. 2010;31(7):832-841. doi:10.1093/eurheartj/ehp458.

28. Matsue Y, Damman K, Voors AA, et al. Time-to-Furosemide Treatment and Mortality in Patients Hospitalized With Acute Heart Failure. *J Am Coll Cardiol*. 2017;69(25):3042-3051. doi:10.1016/j.jacc.2017.04.042.

29. Teixeira A, Arrigo M, Tolppanen H, et al. Management of acute heart failure in elderly patients. *Arch Cardiovasc Dis*. 2016;109(6):422-430. doi:10.1016/j.acvd.2016.02.002.

30. Christ M, Mueller C. Editor’s Choice- Call to action: Initiation of multidisciplinary care for acute heart failure begins in the Emergency Department. *Eur Heart J Acute Cardiovasc Care*. 2016;5(2):141-149. doi:10.1177/2048872615581501.

31. Januzzi JL, Felker GM. Door-to-Furosemide Therapy in the ED: New Quality Metric or Just a Piece of the Puzzle? *J Am Coll Cardiol*. 2017;69(25):3052-3054. doi:10.1016/j.jacc.2017.05.009.

32. Rivers E, Nguyen B, Havstad S, et al. Early goal-directed therapy in the treatment of severe sepsis and septic shock. *N Engl J Med*. 2001;345(19):1368-1377. doi:10.1056/NEJMoa010307.

33. Stevenson EK, Rubenstein AR, Radin GT, Wiener RS, Walkey AJ. Two decades of mortality trends among patients with severe sepsis: a comparative meta-analysis*. *Crit Care Med*. 2014;42(3):625-631. doi:10.1097/CCM.0000000000000026.

34. Zannad F, Garcia AA, Anker SD, et al. Clinical outcome endpoints in heart failure trials: a European Society of Cardiology Heart Failure Association consensus document. *Eur J Heart Fail*. 2013;15(10):1082-1094. doi:10.1093/eurjhf/hft095.

35. Cleland JGF. How to assess new treatments for the management of heart failure: composite scoring systems to assess the patients’ clinical journey. *Eur J Heart Fail*. 2002;4(3):243-247.

36. Cleland JGF, Charlesworth A, Lubsen J, et al. A comparison of the effects of carvedilol and metoprolol on well-being, morbidity, and mortality (the “patient journey”) in patients with heart failure: a report from the Carvedilol Or Metoprolol European Trial (COMET). *J Am Coll Cardiol*. 2006;47(8):1603-1611. doi:10.1016/j.jacc.2005.11.069.

37. Ariti CA, Cleland JGF, Pocock SJ, et al. Days alive and out of hospital and the patient journey in patients with heart failure: Insights from the Candesartan in Heart failure: Assessment of Reduction in Mortality and morbidity (CHARM) program. *Am Heart J*. 2011;162(5):900-906. doi:10.1016/j.ahj.2011.08.003.

38. Allen LA, Hernandez AF, O’Connor CM, Felker GM. End points for clinical trials in acute heart failure syndromes. *J Am Coll Cardiol*. 2009;53(24):2248-2258. doi:10.1016/j.jacc.2008.12.079.

39. Felker GM, Anstrom KJ, Adams KF, et al. Effect of Natriuretic Peptide-Guided Therapy on Hospitalization or Cardiovascular Mortality in High-Risk Patients With Heart Failure and Reduced Ejection Fraction: A Randomized Clinical Trial. *JAMA*. 2017;318(8):713-720. doi:10.1001/jama.2017.10565.

40. Roffi M, Patrono C, Collet J-P, et al. 2015 ESC Guidelines for the management of acute coronary syndromes in patients presenting without persistent ST-segment elevationTask Force for the Management of Acute Coronary Syndromes in Patients Presenting without Persistent ST-Segment Elevation of the European Society of Cardiology (ESC). *Eur Heart J*. 2016;37(3):267-315. doi:10.1093/eurheartj/ehv320.

# addenda

1) list of investigators

| **Name** | **First Name** | **City** | **Country** | **Hospital** | **Expected recruitment** |
| --- | --- | --- | --- | --- | --- |
| Freund | Yonathan | Paris | France | Pitié-Salpêtrière | 34 |
| Adnet | Frederic | Paris | France | AVC | 34 |
| Yordanov | Youri | Paris | France | Saint-Antoine | 34 |
| Feral | Anne-Laure | Paris | France | HEGP | 34 |
| Laribi | Said | Tours | France | CHU Tours | 34 |
| Claret | Pierre-Geraud | Nîmes | France | CHU Nîmes | 34 |
| Chouihed | Tahar | Nancy | France | CHU Nancy | 34 |
| Charpentier | Sandrine | Toulouse | France | CHU Rangueuil | 34 |
| Truchot | Jennifer | Paris | France | Lariboisiere | 34 |
| Dumas | Florence | Paris | France | Cochin | 34 |
| Occelli | Celine | Nice | France | CHU Nice | 34 |
| Khellaf | Mehdi | Creteil | France | CHU H Mondor | 34 |
| Beaune | Sebastien | Boulogne | France | CHU A Paré | 34 |
| Ganansia | Olivier | Paris | France | CH St Joseph | 34 |
| Desmettre | Thibaut | Besancon | France | CHU Besancon | 34 |

2) Model for informed consent

***
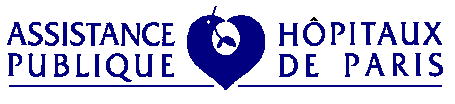
***

**Evaluation d’une prise en charge précoce du syndrome d’insuffisance cardiaque aigüe du sujet âgé aux Urgences : étude randomisée en cluster de type stepped wedge**

**ELISABETH**

**Cette recherche est promue par** **l’Assistance Publique - Hôpitaux de Paris**

**Délégation à la Recherche Clinique et à l’Innovation**

**1 avenue Claude Vellefaux**

**75010 Paris**

**NOTE D’INFORMATION Patient – Version 1-0 du 17/04/2018**

**Madame, Monsieur,**

Le Docteur / Le Professeur ……………………………………. (nom, prénom), exerçant à l’hôpital ……………………………………………., vous propose de participer à une recherche concernant votre état de santé.

Il est important de lire attentivement cette note avant de prendre votre décision ; n’hésitez pas à lui demander des explications.

Si vous décidez de participer à cette recherche, un consentement écrit vous sera demandé.

1. **Quel est le but de cette recherche ?**

Cette recherche porte sur l’insuffisance cardiaque aiguë. Ce syndrome est associé à un risque d’hospitalisation prolongée ou de réhospitalisation à court terme. Peu d’études scientifiques ont pu jusqu’ici mettre en évidence un réel bénéfice à l’application d’une prise en charge ou d’un traitement pour cette pathologie.

Le but de cette recherche est d’évaluer les bénéfices d’une prise en charge médicale complète et précoce de cette affection chez les patients de plus de 75 ans.

Pour répondre à la question posée dans la recherche, il est prévu d’inclure 500 patients présentant une insuffisance cardiaque aigüe, dans des établissements de soins, parmi plusieurs services d’Urgences en France.

1. **En quoi consiste la recherche ?**

Actuellement, les recommandations de prise en charge de l’insuffisance cardiaque incluent l’administration de diurétiques à faible dose, dérivés nitrés à haute dose ainsi que la recherche et le traitement de tout facteur déclenchant. La recherche consiste à vérifier le bénéfice de l’application stricte et précoce de ces recommandations.

De nombreuses études ont précédement pointé la faible adhérence des médecins à ces recommandations, probablement par manque de preuves suffisantes. Une incertitude persiste quant au bénéfice de l’application de ces recommandations.

Dans la recherche proposée, nous allons évaluer une stratégie précoce de prise en charge médicale complète de l’insuffisance cardiaque aiguë comprenant le traitement précoce des symptômes ainsi que, la détection et le traitement de ses potentiels facteurs déclenchant. Pour ce faire, nous allons comparer deux périodes successives d’étude : la première pendant laquelle les patients seront pris en charge de manière habituelle, et où votre médecin pourra ne pas appliquer strictement les recommandations internationales s’il ne le juge pas nécessaire ou indiqué. Dans la seconde période de l’étude, tous les patients seront pris en charge selon une stratégie de prise en charge médicale précoce et complète. Dans tous les cas, les recommandations de prise en charge auront été rappelées au médecin qui vous prend en charge.

Le passage de la période contrôle (habituelle) à la période de stratégie thérapeutique précoce se fera successivement dans chaque centre tous les 15 jours. Ainsi, au bout de 34 semaines, tous les centres appliqueront la stratégie testée. Votre médecin vous indiquera dans quelle période vous êtes inclus.

1. **Quel est le calendrier de la recherche**

La durée prévisionnelle de la recherche est de 40 semaines et votre participation sera de 30 jours après la signature de votre consentement, lors de la première visite. Outre l’application ou non d’une stratégie thérapeutique précoce, un contact téléphonique à 30 jours sera la seule procédure ajoutée par la recherche par rapport à votre prise en charge habituelle.

1. **Quels sont les bénéfices liés à votre participation**

Les bénéfices escomptés de notre stratégie de prise en charge médicale sont une diminution du temps d’hospitalisation par un retour plus précoce au domicile ainsi qu’une diminution du risque de ré-hospitalisation et de la mortalité précoce. L’inclusion pendant la période d’évaluation de notre stratégie pourra être associée à une amélioration du pronostic de cette affection, en particulier d’un retour plus précoce au domicile et d’une diminution du risque de ré-hospitalisation. Par ailleurs, vous contribuerez à une meilleure connaissance de la prise en charge thérapeutique à appliquer pour traiter le syndrome d’insuffisance cardiaque aigüe aux urgences.

1. **Quels sont les risques et les contraintes prévisibles ajoutés par la recherche?**

Dans cette étude, le médecin appliquera la même prise en charge à tous les patients selon la période de l’étude. Dans tous les cas, seuls les traitements habituels et recommandés vous seront administrés, et les procédures diagnostiques habituelles réalisées.

Si vous acceptez de participer, vous devrez respecter les points suivants :

- Suivre les recommandations de votre médecin relatives à votre participation à l’étude.
- Informer le médecin de la recherche, de l’utilisation de tout traitement ainsi que de tout événement survenant pendant la recherche (nouvelle hospitalisation en particulier).
- Ne pas prendre part à un autre projet de recherche sans l’accord de votre médecin, ceci pour vous protéger de tout accident possible pouvant résulter par exemple d’incompatibilités possibles entre les thérapeutiques étudiées ou d’autres dangers pendant 30 jours.
- Etre affilié(e) à un régime de sécurité sociale ou être bénéficiaire d’un tel régime.

1. **Quels sont les éventuelles alternatives médicales ?**

En cas de refus de participer, vous bénéficierez du traitement correspondant à la période de stratégie thérapeutique appliquée dans le centre lors de votre prise en charge . Vous ne serez donc pas inclus dans l’étude,, vos données ne seront pas prises en compte dans cette étude, et nous ne vous recontacterons pas.

1. **Quelles sont les modalités de prise en charge médicale à la fin de votre participation ?**

Votre médecin pourra décider à tout moment de l’arrêt de votre participation ; il vous en expliquera les raisons.

Après votre participation à l’étude, votre prise en charge médicale ne sera pas modifiée et sera effectuée par votre médecin (médecin généraliste, cardiologue ou gériatre) comme prévu habituellement dans le cadre de votre pathologie.

1. **Si vous participez, que vont devenir les données recueillies pour la recherche**

Dans le cadre de la recherche à laquelle l’AP-HP vous propose de participer, un traitement de vos données personnelles va être mis en œuvre pour permettre d’analyser les résultats de la recherche au regard de l’objectif de cette dernière qui vous a été présenté.

A cette fin, les données médicales vous concernant et les données relatives à vos habitudes de vie, seront transmises au Promoteur ou aux personnes ou sociétés agissant pour son compte, en France. Ces données seront identifiées par un numéro de code et vos initiales. Ces données pourront également, dans des conditions assurant leur confidentialité, être transmises aux autorités de santé françaises.

Les données médicales vous concernant pouvant documenter un dossier auprès des autorités compétentes pourront être transmises à un industriel afin qu’un plus grand nombre de patients puissent bénéficier des résultats de la recherche. Cette transmission sera faite dans les conditions assurant leur confidentialité.

Vos données pourront être utilisées pour des analyses ultérieures dans le même champ de recherche ou des analyses complémentaires à la présente recherche en collaboration avec des partenaires privés ou publics, en France ou à l’étranger, dans des conditions assurant leur confidentialité et le même niveau de protection que la législation européenne.

Vous pouvez retirer à tout moment votre consentement à l’utilisation ultérieure de vos données auprès du médecin qui vous suit dans le cadre de cette recherche.

Pour tout arrêt de participation, les données recueillies précédemment à cet arrêt seront utilisées conformément à la réglementation.

**11) Comment cette recherche est-elle encadrée ?**

L’AP-HP a pris toutes les mesures pour mener cette recherche conformément aux dispositions du Code de la Santé Publique applicables aux recherches impliquant la personne humaine.

L’AP-HP a souscrit une assurance (contrat N° 0100518814033180041) garantissant sa responsabilité civile et celle de tout intervenant auprès de la compagnie HDI–GERLING par l’intermédiaire de BIOMEDICINSURE dont l’adresse est Parc d’Innovation Bretagne Sud C.P.142 56038 Vannes Cedex.

L’AP-HP a obtenu l’avis favorable du Comité de Protection des Personnes pour cette recherche [*indiquer le nom du CPP*] le *[indiquer la date de la séance au format jj /mm /aaaa]*

**12) Quels sont vos droits ?**

Votre participation à cette recherche est entièrement libre et volontaire. Votre décision n’entraînera aucun préjudice sur la qualité des soins et des traitements que vous êtes en droit d’attendre.

Vous pourrez tout au long de la recherche demander des informations concernant votre santé ainsi que des explications sur le déroulement de la recherche au médecin qui vous suit.

Vous pouvez vous retirer à tout moment de la recherche sans justification, sans conséquence sur la suite de votre traitement ni la qualité des soins qui vous seront fournis et sans conséquence sur la relation avec votre médecin. A l’issue de ce retrait, vous pourrez être suivi par la même équipe médicale. Dans ce cas, les données collectées jusqu’au retrait seront utilisées pour l’analyse des résultats de la recherche.

Le fichier informatique utilisé pour cette recherche est mis en œuvre conformément à la règlementation Informatique et Libertés (CNIL - loi 78-17 du 6 janvier 1978 modifiée), Vous disposez d’un droit d’accès et de rectification. Vous disposez également d’un droit d’opposition à la transmission des données couvertes par le secret professionnel susceptibles d’être utilisées dans le cadre de cette recherche et d’être traitées. Ces droits s’exercent auprès du médecin en charge de la recherche qui seul connaît votre identité. Vous pouvez également accéder directement ou par l’intermédiaire d’un médecin de votre choix à l’ensemble de vos données médicales en application des dispositions de l’article L 1111-7 du Code de la Santé Publique.

Votre dossier médical restera confidentiel et ne pourra être consulté que sous la responsabilité du médecin s’occupant de votre traitement ainsi que par les autorités de santé et par des personnes dûment mandatées par l’AP-HP pour la recherche et soumises au secret professionnel.

A l’issue de la recherche et après analyse des données relatives à cette recherche, vous pourrez être informé(e) des résultats globaux en le demandant au médecin qui vous suit dans le cadre de cette recherche

Après avoir lu toutes ces informations, discuté tous les aspects avec votre médecin et après avoir bénéficié d’un temps de réflexion, si vous acceptez de participer à la recherche vous devrez signer et dater le formulaire de consentement éclairé se trouvant à la fin de ce document.

**
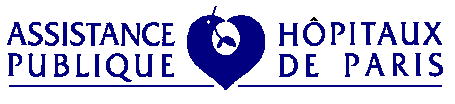
**

**FORMULAIRE DE CONSENTEMENT**

Je soussigné(e), M^me^, M. *[rayer les mentions inutiles]* (*nom, prénom*)………………………………………………………... **accepte librement de participer à la recherche** **intitulée** «  **ELISABETH**  » organisée par l’Assistance Publique - Hôpitaux de Paris et qui m’est proposée par le Docteur/Professeur (*nom, prénom, téléphone*) ……………………………………………………………………………….…, médecin dans cette recherche.

- J’ai pris connaissance de la note d’information version 1-0 du 17/04/2018 (3 pages) m’expliquant l’objectif de cette recherche, la façon dont elle va être réalisée et ce que ma participation va impliquer,

- je conserverai un exemplaire de la note d’information et du consentement,

- j’ai reçu des réponses adaptées à toutes mes questions,

- j’ai disposé d’un temps suffisant pour prendre ma décision,

- j’ai compris que ma participation est libre et que je pourrai interrompre ma participation à tout moment, sans encourir la moindre responsabilité et préjudice pour la qualité des soins qui me seront prodigués.

- j’ai été informé que les données recueillies dans le cadre de la recherche peuvent être réutilisées pour des recherches ultérieures, et que je pouvais m’y opposer à tout moment

- Je suis conscient(e) que ma participation pourra aussi être interrompue par le médecin si besoin, il m’en expliquera les raisons,

- j’ai compris que pour pouvoir participer à cette recherche je dois être affilié(e) à un régime de sécurité sociale ou bénéficiaire d’un tel régime. Je confirme que c’est le cas,

- j’ai bien été informé(e) que ma participation à cette recherche durera 30 jours, et que cela implique que je ne pourrai pas envisager de participer à une autre recherche sans en informer le médecin qui me suit pour la recherche,

- mon consentement ne décharge en rien le médecin qui me suit dans le cadre de la recherche ni l’AP-HP de l’ensemble de leurs responsabilités et je conserve tous mes droits garantis par la loi.

| **Signature de la personne participant à la recherche** | **Signature du médecin** |
| --- | --- |
| Nom Prénom :  Date : Signature : | Nom Prénom :  Date : Signature : |

**Ce document est à réaliser en 3 exemplaires, un exemplaire doit être conservé 15 ans par l’investigateur, le deuxième remis à la personne donnant son consentement et le troisième transmis à l’AP-HP sous enveloppe scellée à la fin de la recherche.**
